# Oligofructose improves small intestinal lipid-sensing mechanisms via alterations to the small intestinal microbiota

**DOI:** 10.1186/s40168-023-01590-2

**Published:** 2023-08-02

**Authors:** Savanna N. Weninger, Chloe Herman, Rachel K. Meyer, Eve T. Beauchemin, Archana Kangath, Adelina I. Lane, Taylor M. Martinez, Tahia Hasneen, Sierra A. Jaramillo, Jason Lindsey, Gayatri Vedantam, Haijiang Cai, Emily K. Cope, J. Gregory Caporaso, Frank A. Duca

**Affiliations:** 1 Department of Physiology, University of Arizona, Tucson, USA; 2 Center for Applied Microbiome Science, Pathogen and Microbiome Institute, Northern Arizona University, Flagstaff, AZ USA; 3 Department of Nutritional Sciences, University of Arizona, Tucson, USA; 4 School of Animal and Comparative Biomedical Sciences, College of Agricultural and Life Sciences, University of Arizona, Tucson, USA; 5 Faculty of Medicine, Department of Microbiology & Immunology, McGill University, Montreal, QC Canada; 6 Department of Neuroscience, University of Arizona, Tucson, AZ USA; 7 Department of Immunobiology, University of Arizona, Tucson, AZ USA; 8 BIO5 Institute for Collaborative Research, University of Arizona, Tucson, USA; 9 Department of Biological Sciences, Northern Arizona University, Flagstaff, AZ USA

**Keywords:** Gut microbiota, Obesity, High-fat diet, Nutrient-sensing

## Abstract

**Background:**

Upper small intestinal dietary lipids activate a gut-brain axis regulating energy homeostasis. The prebiotic, oligofructose (OFS) improves body weight and adiposity during metabolic dysregulation but the exact mechanisms remain unknown. This study examines whether alterations to the small intestinal microbiota following OFS treatment improve small intestinal lipid-sensing to regulate food intake in high fat (HF)-fed rats.

**Results:**

In rats fed a HF diet for 4 weeks, OFS supplementation decreased food intake and meal size within 2 days, and reduced body weight and adiposity after 6 weeks. Acute (3 day) OFS treatment restored small intestinal lipid-induced satiation during HF-feeding, and was associated with increased small intestinal CD36 expression, portal GLP-1 levels and hindbrain neuronal activation following a small intestinal lipid infusion. Transplant of the small intestinal microbiota from acute OFS treated donors into HF-fed rats also restored lipid-sensing mechanisms to lower food intake. 16S rRNA gene sequencing revealed that both long and short-term OFS altered the small intestinal microbiota, increasing *Bifidobacterium* relative abundance. Small intestinal administration of *Bifidobacterium pseudolongum* to HF-fed rats improved small intestinal lipid-sensing to decrease food intake.

**Conclusion:**

OFS supplementation rapidly modulates the small intestinal gut microbiota, which mediates improvements in small intestinal lipid sensing mechanisms that control food intake to improve energy homeostasis.

Video Abstract

**Supplementary Information:**

The online version contains supplementary material available at 10.1186/s40168-023-01590-2.

## Background

The prevalence of obesity and its associated comorbidities has risen dramatically over the last two decades, due in part to the increased palatability of the western diet, high in fat and simple sugars and low in fiber, leading to hyperphagia and positive energy balance [1]. Accumulating evidence suggests that the gut microbiota impacts host metabolic homeostasis [2]. High fat feeding is associated with dysbiotic shifts in the gut microbiota that can contribute to the development of obesity [2, 3]. These shifts occur rapidly in response to a change in diet and can negatively influence metabolism and glucose homeostasis following only a few days of feeding [4, 5]. Conversely, beneficial shifts to the gut microbiota via prebiotics, substrates that are selectively utilized by host microorganisms conferring a health benefit, can lead to improvements in metabolic disease [6, 7]. For example, oligofructose (OFS), an inulin-type fructan, increases the prevalence of beneficial bacteria including *Akkermansia muciphila* and *Bifidobacterium*, and is associated with reductions in food intake, body weight, adiposity, and improvements in glucose homeostasis in both rodents and humans [8–16]. Although not fully understood, the beneficial effects of OFS treatment have been attributed to reduced metabolic endotoxemia due to increased gut barrier integrity, as well as increased enteroendocrine cells (EECs) density and gut peptide secretion [9, 10, 17, 18]. More specifically, OFS treatment increases secretion of glucagon-like peptide-1 (GLP-1), a gut peptide released from EECs in response to nutrients, which is a known incretin and can also lower food intake [19–21]. In fact, glucagon-like peptide-1 receptor (GLP-1R) signaling is required for OFS-mediated improvements in glucose tolerance, but whether this is also true for control of food intake is unknown [8, 13]. Despite OFS improving metabolic regulation and robustly altering the distal gut microbiota and physiology, it must first pass the upper small intestine where it could impact the small intestinal (SI) microbiota. However, to date, no study has examined the role of the small intestine or SI microbiota in mediating the metabolic benefits of OFS treatment.

In addition to nutrient absorption, during a meal, the small intestine senses nutrients and initiates a gut-brain negative feedback loop that controls food intake. For example, in healthy rodents and humans, SI lipids activate a vagal gut-brain signaling mechanism involving release of gut peptides from EECs, and subsequent activation of vagal afferent neurons to control food intake during a meal [22, 23]. However, the suppressive effects of intestinal lipids are abolished during high-fat (HF)-feeding, leading to hyperphagia [5, 24, 25]. This impairment in nutrient-induced negative feedback is associated with reduced gut peptide release and subsequent vagal activation [25, 26]. Although it is not completely understood, recent work has highlighted the potential role of intestinal microbe-host crosstalk in mediating the dietary adaptations in SI nutrient sensing mechanisms.

The gut microbiota can impact intestinal nutrient sensing mechanisms that influence energy homeostasis. For example, germ-free (GF) mice have altered expression of intestinal nutrient receptors and circulating gut peptides compared to conventional controls with a diverse microbiota [27], while conventionalization of GF mice alters SI expression of many genes involved in glucose and lipid metabolism [28]. Additionally, recent work has highlighted the impact of the SI microbiota on the intestinal epithelial chemosensory machinery that mediates a nutrient-induced gut-brain-liver signaling mechanism to regulate hepatic glucose production [5, 24, 29]. For example, HF diet abolishes the ability of small intestinal glucose to lower hepatic glucose production and is associated with a shift in the SI microbiota composition, a decrease in GLP-1 secretion, and reduced intestinal SGLT-1 protein expression which mediates GLP-1 release [29]. However, transplant of the SI microbiota from metformin-treated rats to HF-fed recipient rats restored SI glucose sensing, via increasing SGLT-1 proteins levels and GLP-1 release [29]. Despite these recent findings on the glucoregulatory impact of the SI microbiota, the ability of OFS to restore SI nutrient sensing mechanisms that control food intake has never been explored. Additionally, no studies have examined whether OFS can alter the SI gut microbiota. The current study demonstrates that OFS rapidly alters the SI gut microbiota to restore the SI lipid-sensing and signaling mechanisms that regulate food intake, and identify *Bifidobacterium pseudolongum* as a potential probiotic to increase satiation.

## Methods

### Rats

8–11-week-old male Sprague–Dawley (SD) rats were purchased from Charles River Laboratories (Wilmington, MA). Rats were cohoused (2 rats/ cage) and maintained on a 12-h light/dark cycle with ad libitum access to chow (Teklad Diet #2018), high fat diet (HF, Research Diets D42151), or high fat diet with oligofructose (HF-OFS, Research Diets D19112708; BENEO, Orafti P95), which is macronutrient and calorie matched to the HF diet with the addition of 10% OFS (Supplementary Table 1). Male rats were chosen, as they readily develop obesity when placed on a HF diet and do not have an estrus cycle, which can influence food intake [30–32]. Body composition was measured by quantitative magnetic resonance imaging using EchoMRI-1100 (EchoMRI, Houston, Texas), and rats were placed in metabolic cages (Sable Systems International, Las Vegas, NV, USA) for up to 10 days to record food intake and metabolism.

### Metabolic cage monitoring and indirect calorimetry

11-week-old SD rats (370 ± 12 g; *n* = 8 per group) were placed on a HF diet for 4 weeks prior to being placed in Promethion Core metabolic monitoring cages housed in an environmental chamber maintained at 22 °C and 40% humidity on a 12-h light/dark cycle. Metabolic cages use indirect calorimetry to measure respiratory exchange ratio (RER) and energy expenditure (Weir equation), while continuously monitoring water and food intake. After three days of acclimation, half of the rats were switched to the HF-OFS diet and indirect calorimetry and energy metabolism were recorded for 1 week. Rats were returned to the metabolic cages after an additional 5 weeks of feeding on their respective diets.

### Intestinal contents and tissue collection for gut microbiota and western blot analysis following long-term OFS treatment

A naive group of 8-week-old SD rats (312 ± 21 g; *n* = 10/ group) were used for 6-week microbiota and western blot analysis. Rats were maintained on a HF diet for four weeks before a subset was supplemented with OFS (50 g/ L) in their drinking water for an additional 6 weeks. The concentration of OFS (50 g/l) was chosen based on a pilot study of rats of similar body weight with OFS in water accounting for ~ 8–10% of (w/w) total intake and significantly reducing kilocalorie intake (data not shown). Rats were anesthetized via i.p. injection of ketamine (8 mg/kg) and xylazine (12 mg/ kg), and luminal contents and mucosal scrapings from the duodenum (DUO), jejunum (JEJ), ileum (IL), and cecum (CEC) were collected and snap frozen for microbiota analysis and western blot analysis, respectively.

### Germ-free studies

Male C57BL/6 J germ-free mice (29 ± 2 g; *n* = 9 per group) bred in the University of Arizona Gnotobiotic facility (University of Arizona, Tucson AZ) were switched to a double irradiated HF diet (Research Diets D42151) for 9 days prior to inoculation and maintained on the HF diet until the end of study. Inoculum was prepared as described previously [24, 29]. Prior to inoculation, donor rats were maintained on the same experimental timeline as indicated above (4 weeks HF diet-feeding, followed by 6 weeks of HF-feeding with or without OFS supplementation in drinking water), and fasted 4–6 h prior to transplantation. Rats were anesthetized and the upper portion of the small intestine (15–20 cm; beginning ~ 2–3 cm distal to the SI catheter) was removed, and contents quickly emptied into 50 mL conical tube with 3–4 mL of PBS using sterile procedures. The tube was vortexed and mixture was filtered twice through a 70 µm cell strainer [24, 29]. Mice were inoculated with 200µL of the SI contents of either HF-fed or HF-OFS treated donor rats (2–3 mice per rat). Body weight was measured weekly and epididymal fat was collected at the end of 3 weeks.

### Nutrient-induced satiation studies

10-week-old male SD rats (322 ± 20 g; *n* = 6–8/group) were used to determine the impact of OFS on SI nutrient-induced satiation. Rats were maintained on chow or HF diet for 4 weeks, prior to surgical insertion of an upper SI catheter ~ 6 cm distal to the pyloric sphincter in the upper portion of the jejunum. Following a 5-day recovery, rats were trained on the fasting-refeeding protocol, in which, following an overnight fast, a 15-min SI infusion (0.2 ml/min) of saline, 20% Intralipid (6 kcal; Sigma) or a liquid meal (6 kcal, Ensure®) was given via the SI catheter. During fasting and testing, OFS water was replaced with plain drinking water. Following SI infusion, rats were placed back into their home cages, provided a preweighed amount of food and allowed to eat ad libitum for 2 h, and food intake recorded. SI infusion of Ensure or Intralipid was bracketed by saline infusions, with 2–3 days in-between testing, to determine percent change of food intake from baseline food intake of the saline infusion. A subset of the HF rats was then supplemented with OFS in their drinking water (50 g/L) for 2–3 days and the experiment was repeated).

### Terminal experiment and perfusion

Following nutrient-induced satiation testing, rats were subject to a terminal intestinal lipid-infusion and perfusion study, and additionally, a second group of rats (307 ± 14 g; *n* = 14 total), not tested for nutrient-induced satiation, but following the same diet and surgical timeline was also used. For the terminal experiment, rats were fasted overnight, and one hour following a 15-min upper SI infusion of saline or Intralipid (6 kcal; 20%, Sigma), rats were anesthetized via i.p. injection of ketamine (8 mg/kg) and xylazine (12 mg/ kg). Portal blood was collected in tubes containing DPP-IV inhibitor for GLP-1 analysis and rats were perfused transcardially with ~ 150 mL phosphate-buffered saline (PBS) followed by ~ 150 mL 4% paraformaldehyde. The brain was harvested and fixed in 4% paraformaldehyde for 24 h at 4 °C prior to sectioning and c-Fos staining of the nucleus tractus solitarius (NTS; see below).

### Small intestinal microbiota transplant studies

10-week-old SD rats (327 ± 31 g; *n* = 6–7/group) were used to determine the impact of OFS-induced shifts in the SI microbiota on SI nutrient-induced satiation [24, 29]. Rats were maintained on HF diet for 5 weeks, prior to surgical insertion of an upper SI catheter ~ 6 cm distal to the pyloric sphincter in the upper portion of the jejunum. Following a 5-day recovery, rats were trained on the fasting-refeeding protocol (described above) for nutrient-induced satiation testing (*n* = 6–7/ group) with a subset supplemented with 10% OFS in drinking water for 3 days while the rest remained on HF diet. One day prior to nutrient-induced satiation, rats were subject to SI microbiota transplant (2 recipients per donor rat), similar to previously established protocol [24, 29]. Donor rats were maintained on the same experimental timeline as indicated above (6-week HF rats either supplemented with OFS in drinking water for 3 days or maintained on a HF diet). Donor rats were fasted 4–6 h prior to transplantation, then anesthetized and the upper portion of the small intestine (15–20 cm; beginning ~ 2–3 cm distal to the SI catheter) was removed, and contents quickly emptied into 50 mL conical tube with 3–4 mL of PBS using sterile procedures. The tube was vortexed and mixture was filtered twice through a 70 µm cell strainer, and then 1–1.5 mL was infused directly into SI catheter of recipient rat, and flushed with 0.2 mL saline to clear the line.

Following SI microbiota transplant, rats were overnight fasted and the following morning, tested for nutrient-induced satiation (described above) with SI Intralipid infusion. For the nutrient-induced satiation study, following the refeeding period, rats were fasted for 5 h, and upper and lower SI mucosal scrapings were collected for western blot analysis. For terminal perfusion studies, another group of rats maintained on identical timeline (322 ± 35 g; *n* = 5 per group) was used, but instead of nutrient-induced satiation testing, following SI microbiota transplant, rats were overnight fasted and the following morning, received an intralipid infusion and were sacrificed 1 h after. Portal blood was collected in tubes containing DPP-IV inhibitor for GLP-1 analysis and rats were perfused and brains collected as described previously.

### Intestinal contents collection for gut microbiota analysis following acute OFS treatment

For 3-day microbiota analysis, 10-week-old SD rats (295 ± 12 g; *n* = 6–7/group) were placed on a HF diet for 6 weeks before a subset was supplemented with OFS in drinking water for 3 days. Following a 5-h fast, upper SI (USI), lower SI (LSI), and cecal luminal contents were collected. USI contents (30 cm) were collected beginning 10 cm distal to the pyloric sphincter, and LSI contents (30 cm) were collected ending 10 cm proximal to the cecum (C).

### *Bifidobacterium* culture and administration

*Bifidobacterium pseudolongum* subsp. *pseudolongum* (ATCC 25526) obtained from ATCC (American type Culture Collection, Rockville, Maryland) was cultured for 48 h in modified reinforced clostridial broth (MRCB; ATCC 2107, Remel Inc., Lenexa, Kansas USA) at 37 °C in an anaerobic chamber, followed by subculturing into fresh MRCB. Cultures were grown to an OD_600_ of 1.00 (10^8^ CFU/ml) and harvested by centrifugation (4,000x*g*, 15 min). Bacterial pellets were resuspended in MRCB containing 30% glycerol (vol/vol), aliquoted into 2 ml cryovials and frozen at -80 °C. Prior to animal administration, frozen stocks were thawed and pelleted by centrifugation (4,000x*g*, 15 min) and supernatant decanted. The pellet was then washed three times with sterile phosphate buffered saline (PBS; Cat. # 14,190,136, Gibco). After the final wash, bacteria were resuspended in 1 ml of sterile PBS to achieve a dosing concentration of 10^8^ CFU/ml. This resuspension was administered directly into the small intestine of unanesthetized HF-fed rats via SI catheter within 5 min of final resuspension.

Prior to treatment, 10-week-old SD rats (327 ± 33 g; *n* = 8/ group) were maintained on HF diet for 4 weeks before surgical insertion of an upper SI catheter ~ 6 cm distal to the pyloric sphincter in the upper portion of the jejunum. Following a 5-day recovery, rats were trained on the fasting-refeeding protocol (described above). A subset of the HF-fed rats was given a daily SI infusion of *B. pseudolongum.* Administration was repeated daily for 3 days prior to nutrient-induced satiation testing with saline and Intralipid. At the end of the study, rats were sacrificed, and portal blood was collected in tubes containing DPP-IV inhibitor for GLP-1 analysis. A second group of rats (313 ± 11 g; *n* = 6/ group), not tested for nutrient-induced satiation, but following the same surgical and diet timeline (daily SI *B. pseudolongum* infusion for 3 days following 4 weeks HF-feeding) was used for terminal perfusion studies. Rats were overnight fasted and the following morning, received an intralipid infusion and were anaesthetized 1 h following the Intralipid infusion, perfused and brains collected as described previously.

### Western blotting

Mucosal scrapings collected from the jejunum of 6-week OFS-treated rats (drinking water) or from USI and LSI of 3-day OFS-treated rats were homogenized using NP40 Lysis buffer (Cat. # FNN0021, Invitrogen) with protease (Cat. # P8340, Sigma) and phosphatase inhibitor (Cat. # P5726, Sigma), centrifuged at 12,000 rpm for 15 min at 4 °C, and the supernatant collected. Samples were then assayed for total protein using a BCA protein assay kit (Thermo Scientific) using the manufacturer’s protocol. Loading sample was prepared with sample buffer containing 6X reducing buffer (Cat. # J61337, Alfa Aesar) and 15–20 μg total protein. The denatured proteins were separated by electrophoresis in 1 × Tris–Glycine SDS electrophoresis buffer (Bio5 Media Facility) using 4–20% gradient (CD36 and GPR40) or 10% (GPR120) polyacrylamide gel (Bio-Rad) under reducing conditions (Bio-Rad electrophoresis system; 150-175 V, 1 h; constant voltage), transferred to nitrocellulose (CD36 and GPR40) or PVDF (GPR120) membrane (Bio-Rad) using the wet transfer system (Bio-Rad; 0.75A, constant ampere), and blocked in 5% milk (Carnation, Instant non-fat dry milk)/1 × TBS-tween (Bio5 Media Facility; 0.1% Tween, Cat. # P1379, Sigma) and incubated overnight at 4 °C with primary antibodies in 5% milk/ 1 × TBS-tween (Mouse CD36, 1:1000, Cat. # MABT399, Millipore; Rabbit GPR40, 1:1000, Cat. # VK312429, Invitrogen; Rabbit GPR120, 1:500, Cat. # PA5-50,973, Invitrogen; Mouse β-actin, 1:5000, Cat. # A1978, Thermo Scientific). Membrane was washed, then incubated with IgG secondary antibodies (1:20,000, Rb anti-Ms, Cat. # ab6728, Abcam; Goat anti-Rb, Cat # ab6721, Abcam) for 1 h at room temperature. Protein signals were detected by West Femto Enzyme substrate complex (1:1 ratio, Thermo Scientific) and imaged using the Azure 600 Imager.

### Intestinal catheterization surgeries

Surgical procedures were performed 5–7 days prior to testing. Rats maintained on chow diet were anesthetized via isoflurane inhalation. An upper SI catheter was inserted ~ 6 cm distal to the pyloric sphincter in the upper portion of the jejunum. Subcutaneous injections of meloxicam (1 mg/kg) and saline (5 mL) were given pre-op and two days post-op. Following surgery, rats were individually housed and maintained on a 12-h light/ dark cycle with food intake and body weight monitored daily to ensure rodent recovery.

### Immunohistochemistry

The hindbrain was isolated and sectioned into 100 µm pieces using a vibratome (Leica, VT1000S), and sections were placed in a 24-well plate and stained for c-Fos. Sections were incubated with primary c-Fos antibodies (Rabbit,1:250, Cat. # AB190289, Abcam or Guinea pig, 1:500, Cat# 226,308, SYSY) in PBS with 0.1% Triton X-100 (PBST) overnight at 4 °C. Following incubation, sections were washed 3–4 times with PBST for 5 min and incubated with fluorophore conjugated secondary antibodies (Alexa Flour 594, 1:500, Cat. # 150,080, Abcam or Alexa Flour 488,1:500, Cat# 106–545-003, Jackson ImmunoResearch Labs) in PBST for one hour and wash repeated with PBS. Following addition of ProLong Gold Antifade Mountant (Cat. # P36930, ThermoFischer), tissue sections were observed under a light microscope (ZEISS AxioZoom V16 Fluorescent Microscope) at 63X and 150X magnification and the right and left NTS analyzed for c-Fos positive cells with the help of brain atlas. Analysis was performed for each area by manual counting of c-Fos active cells in two microscopic fields (right NTS and left NTS) per brain slice at 63X from two individuals blinded to experimental conditions and average taken.

### Biochemical analysis

Portal GLP-1 levels were measured using GLP-1 (Active) ELISA (Cat. # EGLP-35 K, Millipore).

### DNA extraction and 16S rRNA gene sequencing

DNA was extracted from intestinal contents using the MagMAX Pathogen RNA/DNA kit (Thermofisher) on the Kingfisher Flex System (ThermoFisher) per manufacturer with an additional lysis step. Prior to extraction, samples were lysed using the supplied bead tubes (Lysing Matrix E, 2 mL tubes) on a vortexer (Fast Prep 24 Homogenizer, MP Bio) on the manufacturer set speed for feces (6 m/s for 40 s). Extractions were performed in a Class II Biosafety Cabinet using protocols adopted from eukaryotic cell culture to protect the samples from contamination (i.e., decontaminate all materials with 70% EtOH to bring into the BSC, double glove while in the BSC, and don single use PPE while working in the BSC). The barcoded primers 515F/806R (Earth Microbiome Project) were used to target the V4 region of the 16S rRNA gene as previously described [33]. Each PCR reaction contained 2.5 µl of PCR buffer (TaKaRa, 10 × concentration, 1 × final), 1 µl of the Golay barcode tagged forward primer (10 µM concentration, 0.4 µM final), 1 µl of bovine serum albumin (Thermofisher, 20 mg/mL concentration, 0.56 mg/µl final), 2 µl of dNTP mix (TaKaRa, 2.5 mM concentration, 200 µM final), 0.125 µl of HotStart ExTaq (TaKaRa, 5 U/µl, 0.625 U/µl final), 1 µL reverse primer (10 µM concentration, 0.4 µM final), and 1 µL of template DNA. ThermalCycler conditions were as follows, 98 °C denaturing step for 2 min, 30 cycles of 98 °C for 20 s, 50 °C for 30 s, and 72 °C for 45 s, a final step of 72 °C for 10 min. PCR was performed in triplicate for each sample and an additional negative control was included for each barcoded primer. Each amplicon was viewed on a 2% agarose gel (E-gel, Invitrogen). Extraction blank controls were processed through the 16S PCR identically to tissue samples. Barcode primer negative template controls (NTCs) were carried through the agarose gel step. If amplification was present for negative controls, PCR was repeated with a new barcoded 806R primer (Earth Microbiome Project). Following agarose gel, PCR product was quantified using the Qubit dsDNA High Sensitivity Kit (ThermoFisher) and the Qubit fluorometer 4 (Invitrogen). PCR products were pooled at equimolar concentrations of 50 ng. The amplicon pool was cleaned using SPRI beads to remove primer dimer (Beckman Coulter). Quality of the pool was assessed with the Bioanalyzer DNA 1000 chip (Agilent Technologies) then combined with 1% PhiX, a bacteriophage often spiked into amplicon sequencing runs to create diversity at each base position being called to get base frequencies closer to the ideal 25% A, 25%C, 25% G, 25% T ratio that is expected by the Illumina sequencing instruments. Amplicons were sequenced on the Illumina MiSeq using the 600-cycle MiSeq Reagent Kit V3 (Illumina).

### Microbiota analysis

Microbiota sequencing data were analyzed using QIIME 2 2022.2 [34]. QIIME 2 provenance replay (https://github.com/qiime2/provenance-lib) was used to generate a fully reproducible description of our workflow [35]. The data was demultiplexed through the q2-demux plugin [36]. Sequence quality control, definition of amplicon sequence variants (ASVs), and construction of the feature table tallying counts of ASVs per sample was performed using DADA2 through the q2-dada2 plugin [37]. The resulting feature table was split into per-anatomical location (duodenum, jejunum, cecum, or USI, LSI, cecum) feature tables for downstream analyses using the filter-samples action in the q2-feature-table plugin. The 3-day analysis was performed on luminal contents from the upper small intestine (USI; *n* = 12), lower small intestine (LSI; *n* = 14), and the cecum (CEC; *n* = 14). The six-week analysis was performed on luminal contents from the duodenum (DUO; *n* = 19), the jejunum (JEJ; *n* = 20), the ileum (IL; *n* = 20), and the cecum (C; *n* = 14). The germ-free analysis was performed on luminal contents of the entire small intestine (SI; *n* = 16) of inoculated germ-free mice.

Each statistical analysis described was run on all 8 datasets (USI, LSI, CEC, DUO, JEJ, IL, C, and germ-free mice). Faith’s Phylogenetic Diversity index and weighted UniFrac were calculated using the q2-diversity plugin’s core-metrics-phylogenetic after a phylogenetic tree was constructed using q2-alignment’s align-to-tree-mafft-fasttree action [38, 39]. As we did not compare samples across locations, even sampling depths varied based on location to maximize the number of samples and sequences that could be included in each analysis. The sampling depths used for each site were USI (7787 sequences per sample), LSI (31,547), CEC (29,714), DUO (14,389), JEJ (9738), IL (34,680), C (18,354), and germ-free mice (20,578). Rarefaction curves based on Faith’s Phylogenetic Diversity index were generated to confirm that richness was stable around the chosen sampling depth for each location. The significance of beta diversity grouping was determined for weighted UniFrac using q2-diversity beta-group-significance with PERMANOVA and ANOSIM tests [40, 41]. The significance of the alpha diversity analysis was determined for Faith’s Phylogenetic Diversity Index with q2-diversity alpha-group-significance using the Kruskal–Wallis test. Taxonomic annotation of ASV was assigned with q2-feature-classifier’s classify-sklearn action using a pre-trained Greengenes classifier accessed from the QIIME 2 website on 6 May 2022 [42–44]. A taxonomy bar plot was generated for each dataset at the phylum and genus levels using the q2-taxa plugin. ANCOM-BC were applied to assess differentially abundance phyla and genera using q2-composition’s ancom-bc action [45, 46]. QIIME 2 version 2022.11 was used to run ANCOM-BC because it is not available in previous versions. Correlation of bodyweight and adiposity with the relative abundance of bacterial taxa at the genus level was performed using the Spearman correlation test with SciPy software [47]. NCBI’s BLASTN 2.13.0 + was applied with default settings and the nt database to identify possible species labels for ASVs classified as *Bifidobacterium* [48]. All *Bifidobacterium* ASVs had a 100 percent identity match over 100% of the query sequence to *Bifidobacterium* genus.

### Statistical analysis

Treatments were assigned randomly to animals with no statistical difference in starting body weights or adiposity when measured between groups. Sample sizes were based off of power calculations using similar previous studies [5, 24, 49]. For long-term HF study with OFS treatment, rats that gained in the bottom 15% for weight and adiposity gain were considered obese-resistant and removed from study. Welch’s *t* test was used to analyze differences between two groups in 6wk OFS studies, microbiota transplant studies and *B. pseudolongum* studies. One-way ANOVA with Tukey’s post hoc test was used to analyze more than two groups in the nutrient-induced satiation studies. Two-way ANOVA with Šídák’s or Tukey’s post hoc tests were used to analyze body weight, food intake, and metabolic cage data over time.* p* value < 0.05 was considered statistically significant.

## Results

### Oligofructose treatment decreases food intake, body weight change, and adiposity in rats on a HF diet

**Fig. 1:**
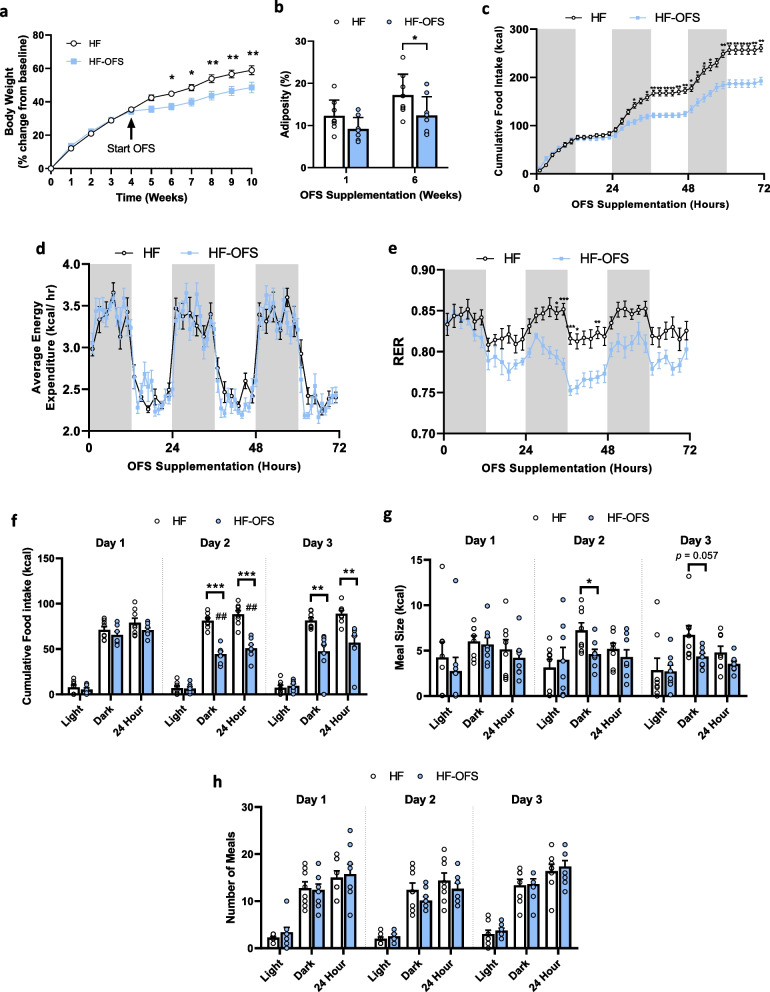
OFS treatment reduces food intake, body weight change, and adiposity in HF-fed rats. Rats were placed on a HF-diet for 4 weeks before a subset was switched to the HF-OFS diet for 6 weeks. **a** 10-week % change in body weight of rats fed a HF (circles) or HF-OFS (squares) diet. **b** Adiposity 1 and 6 weeks after switch to HF-OFS diet or maintenance on HF diet, no difference seen in adiposity prior to start of OFS treatment (data not shown). **c** Cumulative food intake, **d** average energy expenditure, and **e** RER for the first 3 days following switch to HF-OFS diet (squares) or maintenance on HF diet (circles). **f** Cumulative food intake, **g** meal size, and **h** meal number during light, dark, and 24-h time periods over the first three days after switching to HF-OFS diet or maintenance on HF diet. Data in all graphs represent the mean + SEM (*n* = 8 per group); *p < 0.05, **p < 0.01, ***p < 0.001 vs HF-OFS;; #*p* < 0.05, ##*p* < 0.01, ###*p* < 0.001 vs day, as assessed by two-way ANOVA with Šídák's multiple comparisons test or Welch’s *t* test

HF-OFS rats gained significantly less weight over 6 weeks of treatment compared to HF rats (Fig. 1a), despite no significant difference in overall body weight (Supplementary Fig. 1a). The reduced body weight gain observed in HF-OFS rats likely resulted from a reduction in fat mass as these rats had significantly decreased adiposity following 6 weeks of treatment compared to HF rats (Fig. 1b). HF-OFS rats also had significantly reduced percent change in adiposity at both 1 and 6 weeks compared to the HF group (Supplementary Fig. 1b). In line with the early shifts in adiposity and body weight, OFS supplementation reduced cumulative energy intake beginning 32 h after treatment that was maintained for the first week, with no changes in energy expenditure (Fig. 1c-d; Supplementary Fig. 1c-e). Total food intake during the dark cycle was significantly reduced in HF-OFS rats compared to HF rats starting at the second dark cycle, with no differences during any of the light cycles (Fig. 1f). This corresponded with a reduction in meal size during the dark cycle, but no difference in the number of meals consumed in HF-OFS rats after 1 week (Fig. 1g-h; Supplementary Fig. 1f-g), implicating that OFS treatment rapidly induces meal satiation rather than satiety. In addition to reductions in food intake, acute OFS treatment reduced the respiratory exchange ratio (RER) compared to HF rats, denoting that these rats have increased metabolism of fat compared to HF rats (Fig. 1e, Supplementary Fig. 1 h-i). Despite a reduction in adiposity and body weight gain at 6 weeks (Fig. 1b; Supplementary Fig. 1a), no differences in food intake or metabolism were observed between groups at the end of 6 weeks of OFS treatment (Supplementary Fig. 2a-e).

### Long-term oligofructose treatment alters the small intestinal gut microbiota of HF-fed rodents

**Fig. 2:**
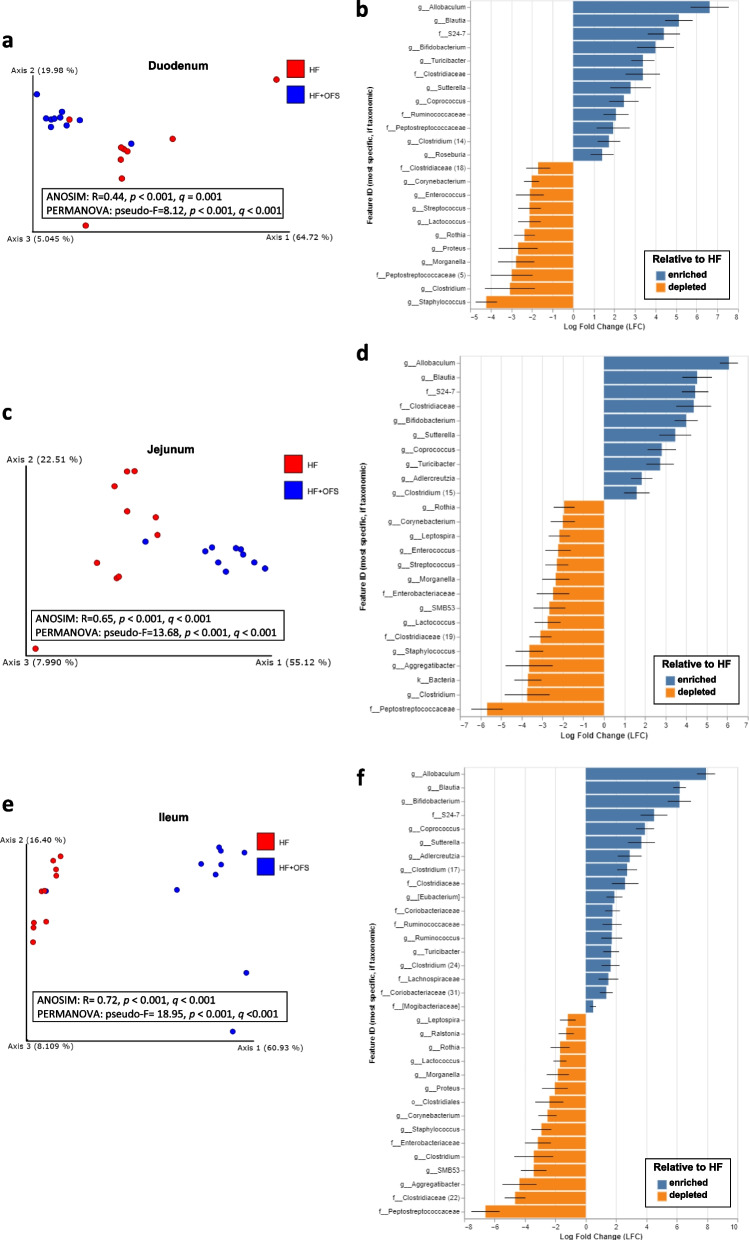
Long-term OFS treatment beneficially shifts the small intestinal microbiota of rats on a HF diet. Analysis of the SI microbiota collected from rats placed on a HF diet for 4 weeks before a subset was supplemented with OFS in their drinking water for an additional 6 weeks. Principal coordinate analysis (PCoA) of weighted UniFrac distances of the **a** duodenal, **c** jejunal, and **e** ileal microbial profiles between HF (red) and HF-OFS (blue) rats. Axes indicate the percentage of variation explained by the plotted principal coordinates. Diverging bar plots show significant Log-Fold Change of bacterial genera in HF-OFS rats compared to HF rats in the **b** duodenum, **d** jejunum, and **f** ileum. Different taxonomic annotation with the same genus label are appended with numbers in order to separate the values. The feature labels (y-axis labels) in each plot represent the most specific named taxonomic level describing the feature. Feature identifiers that are duplicated represent instances of a duplicated taxonomic name at the taxonomic level displayed in the feature identifier. The number following the feature identifiers in these cases is used only for unique identification in the current figure. It is not taxonomically meaningful

To determine whether OFS alters the SI microbiota in HF rats, we performed 16S rRNA gene sequencing of the luminal contents of the duodenum, jejunum, ileum, and cecum following 6 weeks of OFS treatment in HF-fed rats (Fig. 2; Supplementary Fig. 3–5). HF-OFS rats exhibited a unique microbiota profile in each segment of the small intestine, as well as the cecum, compared to untreated HF rats with significant differences in beta diversity (Weighted Unifrac) at each site and alpha diversity (Faith’s PD) in the jejunum and cecum (Fig. 2a,c,e; Supplementary Fig. 4a and 5a-b). At the phylum level, HF-OFS rats exhibited increased *Bacteroidetes* in all segments of the small intestine, as well as increased *Actinobacteria* in the ileum and cecum and decreased *Spirochetes* and *Proteobacteria* in the jejunum and ileum (Supplementary Fig. 4b-d and 5c). ANCOM-BC analysis also revealed significant shifts in several genera and families at each site of the small intestine and the cecum. Specifically, OFS treatment increased prevalence of bacterial genera *Allobaculum*, *Blautia, Bifidobacterium, Sutterella* and *Turcibacter,* and families *S24-7* and *Clostridiaceae* in each segment of the small intestine and the cecum compared to the HF group (Fig. 2b,d,f; Supplementary Fig. 4e-g and 5d-e). Additionally, we observed a decrease in *Corynebacterium, Lactococcus, Rothia, Morganella, Staphylococcus*, and the family *Peptostreptococcaceae* in all sites of the small intestine in the HF-OFS microbiota compared to HF (Fig. 2b, d, f).

To examine the extent to which these changes in the gut microbiota may influence the rat phenotype, we performed a correlation analysis of bodyweight and adiposity with features identified as differentially abundant and with a Log-Fold Change greater than 4 or less than -4 by ANCOM-BC in each segment of the small intestine and cecum of the donor rats. We found that many tested features were significantly correlated with both bodyweight and adiposity, including after correction for multiple comparisons (q-values), at a significance threshold (alpha) of 0.05, which confirms our ANCOM-BC results. In the duodenum, genera *Allobaculum* and *Blautia* and family *S24-7* relative frequencies were negatively correlated, and family *Staphylococcus* relative frequency was positively correlated with adiposity and bodyweight (Supplementary Fig. 6a-e). In the jejunum, the genera *Allobaculum*, *Blautia*, and *Bifidobacterium,* and the families *S24-7* and *Clostridiaceae* were negatively correlated with bodyweight and adiposity (Supplementary Fig. 7a-e). Additionally, the family *Peptostreptococcaceae* relative frequency was positively correlated with body weight (Supplementary Fig. 7f). In the ileum, the genera *Allobaculum*, *Bifidobacterium*, and *Blautia,* and the family *S24-7* relative frequencies were negatively correlated with bodyweight and adiposity, and families *Clostridiaceae* and *Peptostreptococcaceae* were positively correlated with bodyweight and adiposity (Supplementary Fig. 8a-g). In the cecum, *Allobaculum*, *Bifidobacterium*, *Sutterella*, *Clostridium* and *Blautia* relative frequencies were all negatively correlated with bodyweight or adiposity (Supplementary Fig. 9a-e).

**Fig. 3:**
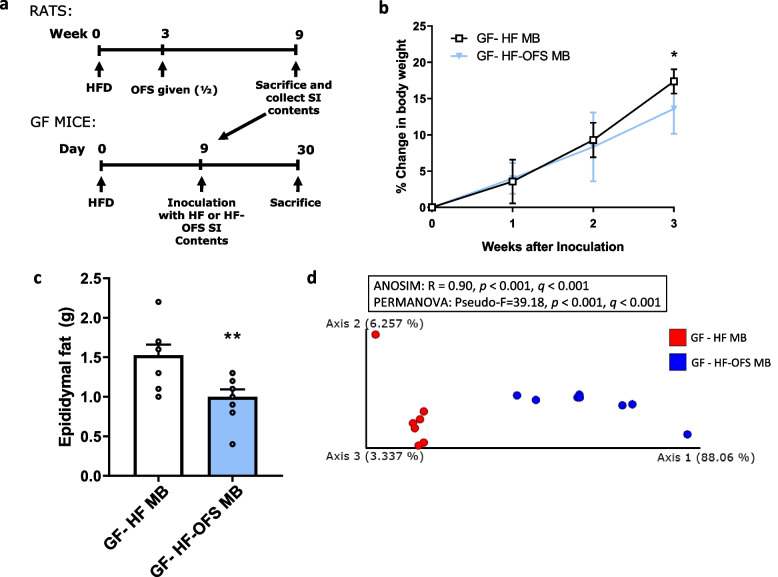
Inoculation of germ-free mice with the gut microbiota from HF-OFS rats decreases bodyweight gain and fat mass compared to HF rats. Inoculation of GF mice with the SI contents from HF-fed rats with or without 6-week supplementation of OFS in drinking water. **a** Experimental timeline. **b** Percent change in body weight following HF-OFS (GF-HF-OFS, triangles, *n* = 9) or HF alone (GF-HF, open squares, *n* = 8) microbiota transplant in GF mice maintained on a HF diet. **c** Epididymal fat content 3 weeks following microbiota transplant from HF (GF-HF, white bar, *n* = 8) or HF-OFS treated (GF-HF-OFS, blue bar, *n* = 9) rats. **d** Principal coordinate analysis (PCoA) of weighted UniFrac distances of the small intestinal microbiota of GF mice 3 weeks following inoculation with the SI microbiota from HF (*n* = 7) or HF-OFS (*n* = 9) treated rats. Data in all graphs represented as mean + SEM; *p < 0.05 (two-way ANOVA with Sidak’s post test), **p < 0.01 (unpaired t test with Welch’s correction)

To determine whether these changes in the SI microbiota following OFS treatment contribute to reductions in adiposity, germ-free mice were inoculated with the SI contents of 6-week HF- or HF-OFS rats. Mice inoculated with the SI microbiota of HF-OFS rats gained less weight and had less epididymal fat at 3 weeks after inoculation compared to recipients of the microbiota from HF rats (Fig. 3a-c). Furthermore, analysis of the SI microbiota of these germ-free mice confirmed an increased abundance of *Allobaculum* in the OFS recipients similar to the donor rats (Supplementary Fig. 10a). There was also a significant Weighted Unifrac distance and Faith Phylogenetic diversity between the OFS recipients and the HF recipients (Fig. 3d; Supplementary Fig. 10b). These data suggest that the OFS treatment results in microbiota shifts of the small intestine, with increased relative abundance of several beneficial bacteria previously known to be increased in the distal intestine, and that these shifts in the SI microbiota may play a causal role in the decrease in adiposity following OFS treatment.

### Short-term oligofructose restores the gut-to-brain signal to improve nutrient-induced satiation, which is abolished with HF-feeding

**Fig. 4:**
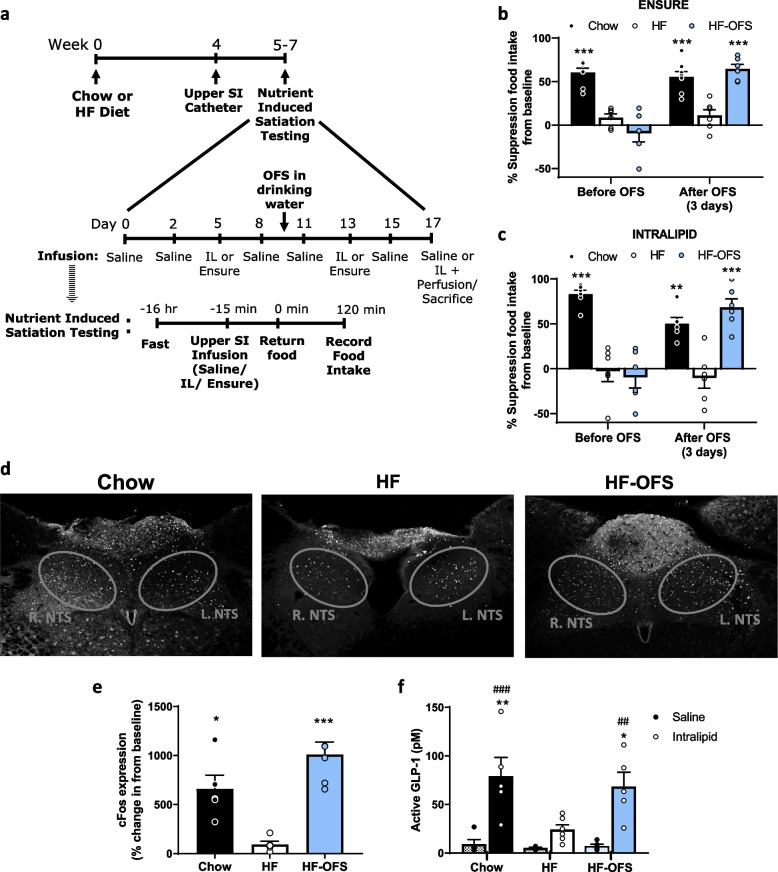
OFS restores SI lipid sensing mechanisms that control food intake in HF-fed rats. Nutrient-induced satiation testing and terminal perfusion studies in rats placed on either a chow or HF diet with or without OFS supplementation in the drinking water. **a** Experimental timeline. **b** and **c** Percent suppression of food intake from baseline saline infusion following SI infusion of **b** Ensure or **c** Intralipid (IL) before and after OFS treatment. **d** Immunohistochemistry and **e** percent change in c-Fos expression in the NTS of chow, HF, and HF-OFS rats 1 h after a 15-min SI infusion of intralipid compared to c-Fos expression following a saline infusion. **f** Portal GLP-1 levels in chow, HF, and HF-OFS 1 h following a 15-min SI infusion of saline (black circles) or intralipid (white circles). Data in all graphs represent the mean + SEM (*n* = 4–6 per group); *p < 0.05, **p < 0.01, ***p < 0.001 vs HF; ##*p* < 0.01, ###*p* < 0.001 vs saline within group, as assessed by one-way ANOVA with Tukey’s post hoc test

**Fig. 5:**
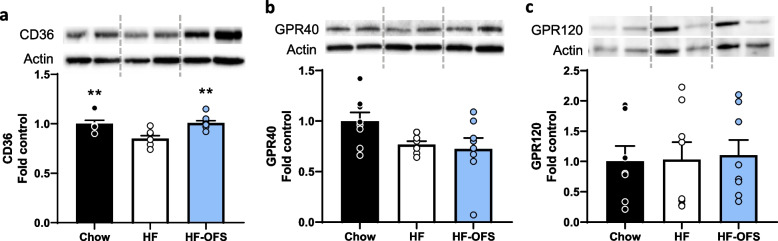
OFS restores jejunal nutrient-sensing proteins. Jejunal mucosal scrapings from rats placed on either a chow or HF diet with or without 6-week OFS supplementation in the drinking water. Relative protein expression of **a** CD36, **b** GPR40, and **c** GPR120 in the jejunum of chow, HF, and HF-OFS rats. Data in all graphs represent the mean + SEM (*n* = 4–6 per group); *p < 0.05, **p < 0.01, ***p < 0.001 vs HF, as assessed by one-way ANOVA with Tukey’s post hoc test

High-fat feeding impairs SI nutrient sensing mechanisms that control food intake via a gut-brain axis [25, 26]. Similar to previous work [26], suppression of food intake following infusion of either Ensure or Intralipid compared to saline control infusion was significantly decreased in HF rats compared to chow-fed rats, indicating an impairment in nutrient-induced satiation during HF-feeding. However, following just three days of OFS supplementation in drinking water, we observed a complete restoration of this nutrient-induced satiation in response to a SI infusion of either Ensure or Intralipid (Fig. 4a-c; Supplementary Fig. 11a-b). This is in line with our metabolic cage data indicating that reductions in meal size are observed after 2–3 days of OFS treatment (Fig. 1g). We initially examined the effect of Ensure, as it is a complete liquid meal, and would complement our data indicating OFS reduces food intake and specifically meal size. However, given that HF-feeding impairs lipid-induced satiation, vagal gut-brain signaling has previously been shown to mediate the suppressive effect of SI lipids [23], and that vagal signaling is diminished following HF-feeding [5, 50], we examined hindbrain activation following a 15-min SI infusion of saline or Intralipid. Complementing the food intake data, HF rats had reduced c-Fos activation in the NTS, where vagal afferents terminate, compared to chow rats following SI Intralipid infusion. However, 3-day supplementation with OFS resulted in a complete restoration of c-Fos in the NTS, potentially indicating that OFS treatment restores lipid-induced vagal afferent signaling that reduces food intake (Fig. 4d-e). Given the previous links between OFS treatment and GLP-1 signaling, and the fact that activation of the GLP-1R on vagal afferents lowers food intake [13, 51], we measured portal GLP-1 concentrations 15 min after either a saline or lipid infusion. Intralipid infusion significantly increased portal GLP-1 concentration in chow and HF-OFS rats, but not HF rats, compared to saline infusion, and GLP-1 levels were significantly higher in HF-OFS rats following lipid infusion compared to HF rats (Fig. 4f). Importantly, these rats were not exposed to OFS during fasting or testing, thus the secretion of GLP-1 was due solely to changes in lipid-sensing mechanisms that induce GLP-1 secretion, and not due to OFS directly. Lipid-induced gut peptide secretion from EECs is mediated via CD36, GPR40, and possibly GPR120 [20, 52, 53]. In line with this, protein expression of CD36 in the jejunum of HF rats was decreased compared to chow, while HF-OFS restored CD36 levels (Fig. 5a; Supplementary Fig. 12a). We found no differences in GPR40 or GPR120 protein expression in the jejunum (Fig. 5a-b; Supplementary Fig. 12a-b).

### Alterations to the upper small intestinal microbiota following OFS supplementation drive the improvements in lipid-sensing mechanisms controlling food intake in rats on a HF diet

**Fig. 6:**
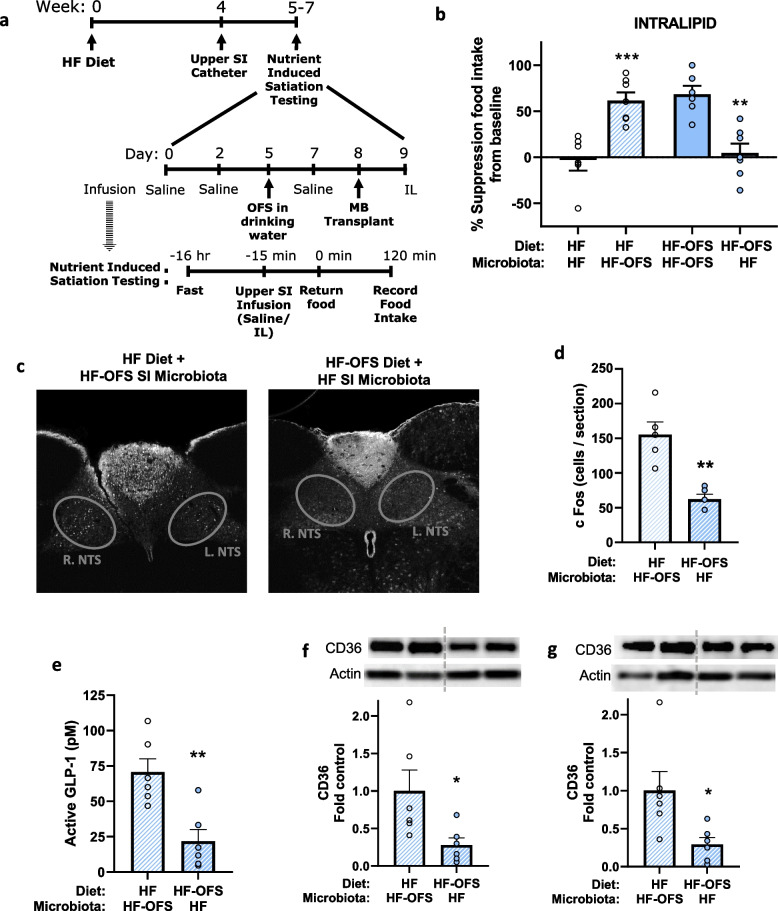
Alterations to the SI microbiota following OFS treatment mediate the improvements in lipid-induced satiation. Nutrient-induced satiation testing and terminal perfusion studies in rats placed on a HF diet with or without OFS in the drinking water and given a SI microbiota transplant from HF or HF-OFS rats. **a** Experimental timeline. **b** Percent suppression of food intake following SI infusion of intralipid from baseline SI saline infusion before and after SI microbiota transplant from HF-OFS rats to HF rats and from HF rats to HF-OFS rats. **c** Immunohistochemistry and **d** quantification of c-Fos expression in the NTS following a 15-min SI infusion of Intralipid following microbiota transplants. **e** Portal GLP-1 levels following a 15 min SI infusion of Intralipid. CD36 protein expression in the **f** USI and **g** LSI following microbiota transplant from HF-OFS rats to HF rats and from HF rats to HF-OFS rats. Data in all graphs represent the mean + SEM (*n* = 6–7 per group); *p < 0.05, **p < 0.01, ***p < 0.001, as assessed by Welch’s *t* test

Given our current findings that OFS treatment alters the SI microbiota, we next sought to determine whether improvements in lipid-induced satiation following OFS treatment are due to the shifts in the SI microbiota. To do this, we performed SI microbiota transplants the day before testing the suppressive effects of a SI lipid infusion (Fig. 6a). Transplant of the SI microbiota from HF-OFS rats to HF rats restored the ability of SI Intralipid infusion to decrease food intake. Conversely, transplant of the SI microbiota from HF-fed rats to HF-OFS rats abolished the suppressive effects of an SI lipid infusion (Fig. 6b; Supplementary Fig. 11c). The restoration and impairment of this nutrient-induced satiation was paralleled by increased and decreased NTS c-Fos expression and portal GLP-1 levels, respectively (Fig. 6c-e). Additionally, we observed increases in upper small intestine (USI) and lower small intestine (LSI) CD36 protein expression in rats that received the SI microbiota from HF-OFS rats compared to HF microbiota recipients (Fig. 6f-g; Supplementary Fig. 13a-b). These results demonstrate that the SI gut microbiota can increase intestinal lipid sensing machinery to improve nutrient-induced gut-brain signaling mechanisms that control food intake, and that OFS restores the ability of SI lipids to lower food intake via changes in the SI microbiota.

### Three days of OFS supplementation alters the small intestinal microbiota composition

**Fig. 7:**
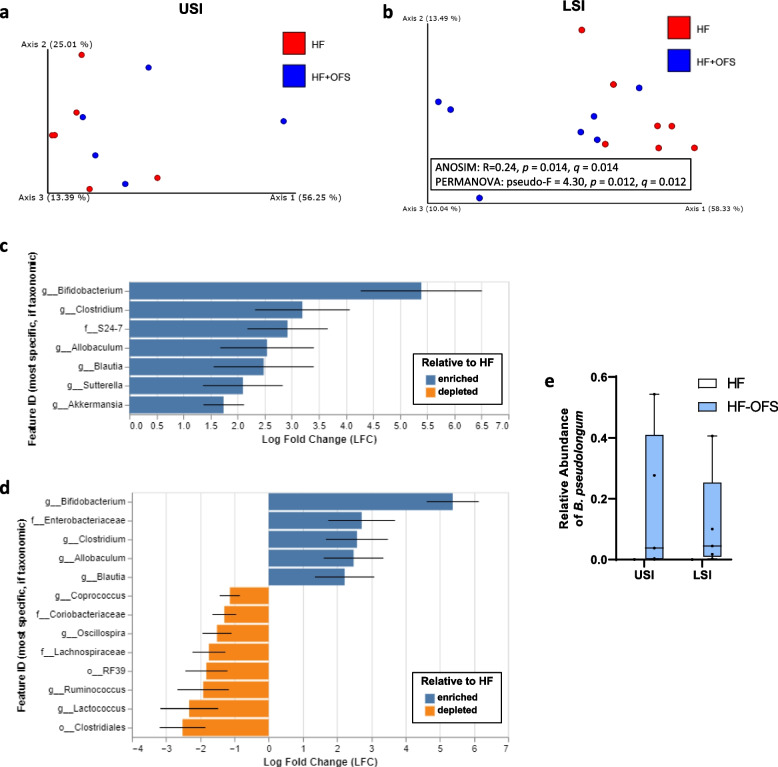
Acute OFS treatment alters the small intestinal microbiota. Rats were fed a HF-diet for 6 weeks and a subset was supplemented with OFS in drinking water for 3 days. Principal coordinate analysis (PCoA) of weighted UniFrac distances of the **a** USI and **b** LSI microbial profiles between HF (red) and HF-OFS (blue) rats. Axes indicate the percentage of variation explained by the plotted principal coordinates. Diverging bar plots show significant Log-Fold Change of bacterial genera in HF-OFS rats compared to HF rats in the **c** USI and **d** LSI. Different taxonomic annotation with the same genus label are appended with numbers in order to separate the values. The feature labels (y-axis labels) in each plot represent the most specific named taxonomic level describing the feature. Feature identifiers that are duplicated represent instances of a duplicated taxonomic name at the taxonomic level displayed in the feature identifier. The number following the feature identifiers in these cases is used only for unique identification in the current figure. It is not taxonomically meaningful. **e** Relative frequency of *Bifidobacterium pseudolongum* in the USI and LSI

Given that our initial data demonstrates that shifts in the SI gut microbiota occur after 6 weeks of OFS treatment, we further analyzed the SI gut microbiota after short-term OFS treatment, mirroring our behavioral studies. Indeed, 3-day OFS treatment significantly altered beta diversity in the LSI, but not USI, and both alpha and beta diversity in the cecum (Fig. 7a-b; Supplementary Fig. 14a,d-e). Phylum level analysis revealed increased *Bacteroidetes* and *Verrucomicrobia* in the USI, increased *Proteobacteria* and *Actinobacteria* in the LSI, and increased *Actinobacteria* in the cecum of HF-OFS treated rats (Supplementary Fig. 14b-c,f). Additionally, we observed increased levels of *Bifidobacterium, Clostridium, Allobaculum,* and *Blautia* in both the USI and LSI as well as the cecum in HF-OFS rats relative to HF-fed rats with ANCOM-BC analysis (Fig. 7c-d; Supplementary Fig. 14 g-h). The USI also exhibited increases in the *Sutterella and Akkermansia genera,* and the family *S24-7* with OFS-treatment (Fig. 7c). In the LSI, HF-OFS rats also had increased *Enterobacteriaceae* and decreased *Coprococcus,, Oscillospiraceae, Ruminococcus, Lactococcus, Coriobacteriaceae, Lachnospiraceae, RF39,* and *Clostridiales* compared to HF-fed rats (Fig. 7d)*.* Our assignments for *Bifidobacterium* included *Bifidobacterium pseudolongum* and *Bifidobacterium animalis*. To confirm whether a specific species of *Bifidobacterium* was increased in the small intestine of OFS treated rats, we searched the ASV sequences assigned to *Bifidobacterium* against the NCBI ntdatabase using default settings. We found significant alignments with the strain confirming the annotations of *Bifidobacterium pseudolongum* and *Bifidobacterium animalis* for all query sequences (100% query coverage and 100% percent identity). *B. pseudolongum* was significantly increased in the USI, LSI, and cecum (Fig. 7e; Supplementary Fig. 14i-l). Given this, we directly tested the effect of *B. pseudolongum* on lipid-induced satiation.

### Small intestinal administration of *Bifidobacterium pseudolongum* restores small intestinal lipid sensing to decrease food intake in HF-fed rats

**Fig. 8:**
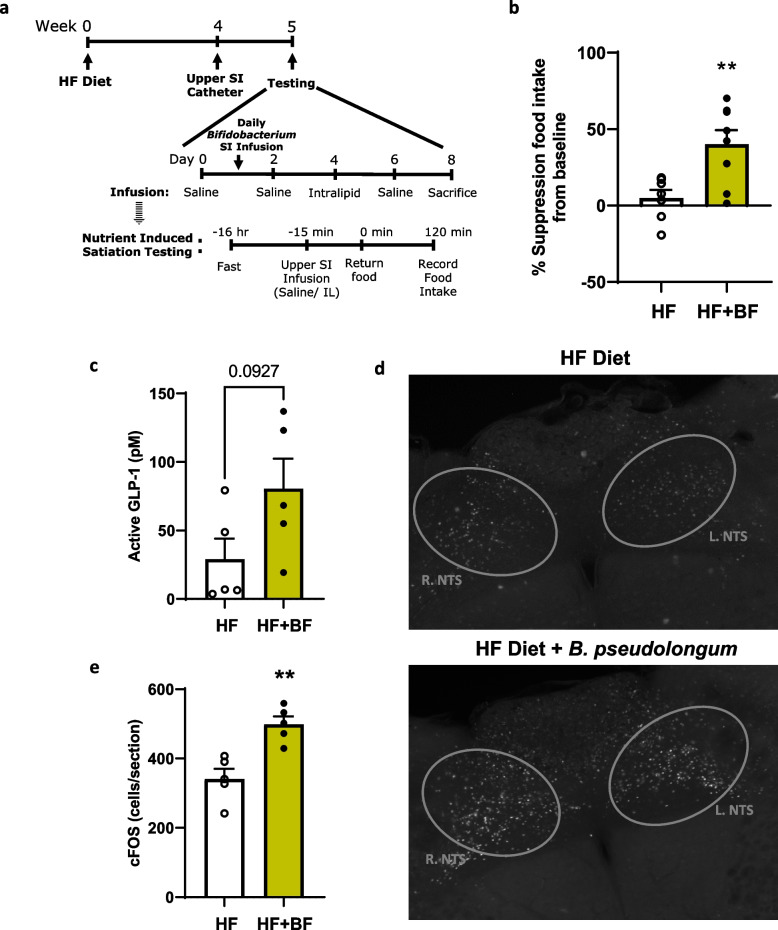
Daily administration of *B. pseudolongum* improves lipid sensing to decrease food intake via a gut-brain signal. Rats were fed a HF diet for 4 weeks prior to daily administration with *B. pseudolongum* via SI catheter for 3–7 days. **a** Experimental timeline. **b** Percent suppression of food intake following SI infusion of intralipid from baseline SI saline infusion in HF and *B. pseudolongum* (BF; 3 days) -treated HF rats. **c** Portal GLP-1 levels at one hour following a 15-min SI infusion of Intralipid in HF or HF-BF rats (7 days). **d** Immunohistochemistry and **e** quantification of c-Fos expression in the NTS following a 15-min SI infusion of Intralipid following *B. pseudolongum* SI administration (3 days). Data in all graphs represent the mean + SEM (*n* = 5–8 per group); **p < 0.01, as assessed by Welch’s t test

Daily administration of cultured *B. pseudolongum* in HF-fed rats for 3 days, significantly reduced food intake following a lipid infusion compared to saline infusion, which was not observed in control HF-fed rats (Fig. 8a-b). Additionally, we observed a slight increase in portal GLP-1 levels with *B. pseudolongum* treatment, although it did not reach significance (Fig. 8c).This was accompanied by increases in NTS activation following a SI lipid infusion in 3-day *B. pseudolongum*-treated rats on a HF-diet (Fig. 8d-e).

## Discussion

The burgeoning appreciation for the role of the gut microbiota in the development of metabolic disease and as a target for the treatment of obesity and metabolic disorders necessitates investigation into anti-obesogenic agents that alter the gut microbiota. Indeed, prebiotic treatment, especially OFS and other inulin-type fructans, reduces body weight and adiposity, which is associated with shifts in the distal gut microbiota in rodents [9, 11–13]. Despite the extensive work demonstrating the impact of the distal gut microbiota in contributing to host energy homeostasis, few studies have examined the metabolic role of the SI gut microbiota. The small intestine plays a vital role in regulating food intake following a meal, in a large part due to the ability of the intestine to sense nutrients and activate a gut-brain axis involving the release of gut peptides from EECs [20]. Similar to previous reports, we demonstrate that increased adiposity and body weight gain during HF-feeding is associated with impaired SI lipid-sensing and activation of a gut-brain axis. Although previous studies examining the beneficial metabolic effects of OFS have demonstrated that decreased adiposity and improved glucose homeostasis was associated with increased circulating gut peptide levels [8, 17], no study has addressed the impact of SI nutrient sensing mechanisms on mediating the metabolic benefits of OFS treatment. Here we show that short-term OFS supplementation in rats on a HF diet restores SI lipid-sensing pathways that control food intake via a gut-brain axis. Furthermore, we find that these improvements in nutrient-induced gut-brain signaling are due to shifts in the SI microbiota. Transfer of the SI microbiota from HF-OFS rats to HF rats was able to recapitulate the ability of OFS treatment to restore the suppressive effects of intestinal lipids on food intake. In line with this, we observed shifts in the SI microbiota after short- (3 day) and long-term (6 week) treatment of OFS, most notably an increase in *Bifidobacterium*. It should be noted that we only examined the effect of OFS on the luminal microbiota, which differs from the mucosal microbiota that may be differentially influenced by OFS administration [54]. Transplant of the SI microbiota from long-term OFS-treated rats into GF mice decreased food intake, bodyweight, and adiposity compared to HF- microbiota GF recipients. However, GF mice exhibit developmental and physiological differences from conventionally raised animals and it is difficult to determine if the effect was due solely to differences in the SI microbiota after transplant or from differences in the distal microbiota [27]. Therefore, we also performed transplant of the SI microbiota from short-term OFS treated or untreated rats into the small intestine of conventional rats, which represents a more physiologically relevant model to test the causality of shifting the SI microbiota. Indeed, shifting the SI microbiota to that of the treated or untreated donor rats recapitulated the effects of OFS and HF-feeding on SI lipid sensing. Lastly, SI administration of *Bifidobacterium pseudolongum*, the primary species of *Bifidobacterium* increased in the short-term OFS treated rats, improved SI nutrient-induced satiation and hindbrain activation independent of OFS. Overall, these studies highlight a novel role of the SI microbiota in mediating the beneficial effects of OFS treatment via improvements in SI lipid-sensing and control of food intake via a gut-brain axis.

Under normal conditions, ingested nutrients trigger a negative feedback loop in the small intestine that controls subsequent food intake. This mechanism involves the release of gut peptides from EECs that likely act on their receptors on nearby vagal afferent neurons [20]. During HF-feeding, both rodents and humans have an impaired response to SI lipids [5, 24, 25]. In rats, HF-feeding results in diminished suppression of food intake, which is associated with reductions in vagal afferent activation measured by both electrophysiology and c-Fos activation in the NTS of the hindbrain [55]. Nutrient stimulated GLP-1 release is also decreased following HF-feeding, and GLP-1R signaling has been shown to mediate the suppressive effects of jejunal lipids [24, 29]. Here we demonstrate that short-term OFS treatment restores HF-induced impairments in lipid sensing, resulting in a suppression of food intake following a lipid infusion, comparable to what is found in healthy chow-fed rodents. We found this is likely due to a combination of increased secretion of GLP-1, as measured in the portal vein, following SI lipid infusion, and a subsequent increase in c-fos activation in the NTS of the hindbrain. However, we cannot rule out the possibility that the effects of OFS were mediated via increases in other gut peptides as well as shifts in the circadian cycle. For example, while GLP-1 mediates nutrient-induced intestinal control of hepatic glucose production, other peptides like CCK and PYY are also known to be released in response to lipids and at least partly mediate effects on food intake [20]. Furthermore, previous studies demonstrate that HF-feeding alters normal diurnal shifts in the microbiota and this rhythm can directly influence GLP-1 secretion, but whether OFS can restore impairments in circadian cycle remains to be explored [56, 57]. We also cannot rule out that the increases in GLP-1 could slow gastric emptying and contribute to the observed reductions in food intake following OFS treatment in conjunction with restoration of gut-brain signaling [58]. Nonetheless, we hypothesize that the increased GLP-1 secretion and subsequent reductions in food intake following a small intestinal lipid infusion was due in part to a restoration of CD36 protein expression in the SI epithelium following OFS treatment and SI microbiota transplant of OFS-treated donor rats, as previous studies demonstrate that knockdown of CD36 results in decreased gut peptide secretion [52]. Interestingly, evidence suggests that CD36 is required for gut peptide release, although not necessarily on the EECs [52]. One hypothesis is that long chain fatty acids enter the enterocyte via CD36 and are packaged into chylomicrons. Upon secretion at the basolateral membrane of the epithelial cells, chylomicrons are broken down and the fatty acids can activate G-protein coupled receptors, GPR40 or GPR120, on the basolateral membrane of nearby EECs to trigger the release of gut peptides [20]. In addition to a lowered absorption of intestinal lipids via CD36, chylomicron formation and secretion is impaired in the small intestine of CD36 knock-out mice [59], highlighting a potential role of CD36 in meditating SI lipid sensing. However, in one of the few other studies that examined the effect of HF-feeding on the SI microbiota, it was found that while a HF diet decreased SI *Bifidobacterium*, jejunal CD36 gene expression was increased in HF-fed mice, although this might be due to slight differences in the diet or species or SPF condition [60]. In contrast, *Bifidobacterium* was significantly increased in each section of the small intestine and the cecum of our 6-week OFS treated rats, and we observed significant increases in jejunal CD36 expression. These discrepancies warrant future investigation to elucidate the specific mechanisms through which alterations in the SI gut microbiota via OFS treatment could increase CD36. The precedent for this is supported by the findings that: 1) GF mice have reduced expression of intestinal CD36 and other nutrient transporters and receptors compared to conventionalized mice [27]; 2) in vitro cell culture and organoid models demonstrate specific bacteria alter expression levels of long chain fatty acid receptors and CD36 [60, 61]; 3) similar to CD36, the glucose transporter SGLT-1 is regulated by the SI gut microbiota [29]. Nonetheless, the improvements in nutrient-induced satiation following OFS treatment was reflected in the observed decreases in meal size following OFS treatment in *ad lib* fed rats as early as two days following treatment, before any changes in adiposity were observed. Importantly, this increase in satiation was not compensated for by an increase in meal number, resulting in an overall decrease in cumulative intake throughout the dark cycle. Thus, it is plausible that improvements in adiposity are due to cumulative reductions in meal size rapidly following OFS supplementation that is a result of a restoration in the SI lipid sensing gut-brain pathway that lowers food intake. The observation that OFS treatment resulted in no difference in food intake or metabolism at the end of 6 weeks, may possibly be due to adaptive mechanisms following substantial adiposity loss back to normal control levels, indicating that early changes in meal satiation drives long-term improvements in energy homeostasis.

The rapid shifts we observed in the SI microbiota and the improvement in lipid-sensing corresponds with the OFS-induced decrease in cumulative food intake and meal size we observed, which began during the second dark cycle. Shifts in the distal gut microbiota occur rapidly after a dietary switch, with the establishment of a newly stabilized gut microbiota after only 2 days in humans and as soon as 18 h in mice [3]. Furthermore, diet-induced shifts in the microbiota occur prior to changes in metabolic outcomes like dysregulated glycemia and is independent of changes in adiposity [4]. Thus, the reduction in food intake and meal size we observed after about 30 h of treatment is likely a result of this rapid shift in the SI microbiota following OFS treatment. In support of this, we observed no difference in the dark cycle food intake in the first day, as the diet had likely not yet shifted the gut microbiota, indicating that decreased food intake is not due to a change in the diet alone.

Oligofructose treatment beneficially alters the distal gut microbiota in both rodents and humans [12, 62]. Similar to previous studies, we found that long-term OFS treatment drastically alters the distal gut (cecum) microbiota, increasing genus level relative abundance of several genes and families including *Bifidobacterium*, *Allobaculum, Blautia*, *Sutterella,* and *S24-7*. Our findings support previously characterized prebiotic-induced increases in the beneficial *Bifidobacterium*, which were associated with increased GLP-1 and gut barrier integrity [9, 12, 17]. Increases in *Allobaculum* with prebiotic treatment has also been reported with both acute and chronic OFS treatment, but its role in improving homeostasis is not well established [63, 64]. However, to our knowledge, this is the first study to demonstrate that OFS alters the SI microbiota and supports a recent human study demonstrating that galacto- and fructo-oligosaccharides are capable of being fermented by the SI microbiota [65]. Importantly, we observed rapid (3 days) improvements in lipid-sensing mechanisms, and transplant of this SI microbiota was able to recapitulate the improvements, highlighting an SI microbiota-mediated effect independent of the fiber-effect of OFS in acute improvements in SI nutrient sensing. Although the transfer of OFS still residing in the small intestine during the transplant could impact these findings, this is unlikely as the amount of OFS would have been extremely low due to a 5 h fast and small amount of luminal contents collected. Additionally, we doubt the effect was due to the potential transfer of OFS as we found *B. pseudolongum* treatment per se was able to improve nutrient sensing and restore NTS activation following a SI lipid infusion. Indeed, although acute OFS treatment only moderately altered the overall SI microbiota composition compared to long-term treatment, possibly due to a lower number of animals or from the aforementioned impact of host phenotype changes on gut microbiota composition, we still observed a significant increase in the relative abundance of *Bifidobacterium*, specifically *Bifidobacterium pseudolongum,* in the upper and lower small intestine, and the cecum. *B. pseudolongum* are Gram-positive, anaerobic bacteria, known to break down nondigestible carbohydrates [66]. Recent studies have demonstrated that *B. pseudolongum* administration decreases food intake, bodyweight, and fat mass in mice on a HF diet, and can improve intestinal barrier integrity [67, 68]. However, this is the first study to demonstrate that *B. pseudolongum* can improve nutrient-sensing mechanisms that control food intake. Although we found an increase in c-Fos activity in the NTS in *B. pseudolongum* treated rats following intralipid infusion, there was only a slight, albeit non-significant, increase in portal GLP-1 concentration. Therefore, it is possible that *B. pseudolongum* improves nutrient sensing through increased release of a combination of gut peptides, like CCK, which is also known to vagally regulate lipid-induced satiation. Nonetheless, we hypothesize that the ability of OFS to improve SI nutrient-sensing mechanisms is due to its ability to increase *Bifidobacterium* levels in the small intestine. Future studies will assess the specific mechanisms through which SI *Bifidobacterium* improves nutrient-sensing, however, *Bifidobacterium* can decrease intestinal inflammation and increase gut barrier integrity [9, 10, 69]. Intestinal inflammation is associated with impaired lipid absorption [70], thus it is possible that increased SI *Bifidobacterium* acts to reduce SI inflammation and increase CD36 levels that ultimately increase lipid absorption. Furthermore, OFS improvements in barrier integrity are at least partly mediated by the gut peptide glucagon like peptide-2 (GLP-2) [10]. Interestingly, GLP-2 increases SI lipid absorption in a CD36-dependent manner and enhances the release of chylomicrons from enterocytes, which can act on EECs to stimulate GLP-1 release [71, 72]. Given that OFS increases circulating GLP-2 levels [10], and that we observed increases in GLP-1 which is secreted by the same L-cells, future studies will elucidate the exact mechanisms of how SI *Bifidobacterium* can increase CD36 protein expression, possibly via increased GLP-2 signaling.

## Conclusions

Overall, we have established that alterations in the SI microbiota following OFS treatment improves SI nutrient sensing mechanisms that control food intake via a gut-brain axis. These improvements in nutrient-induced neuronal feedback resulted in reductions in meal size that was associated with reduced adiposity. Specifically, we highlight a role for *Bifidobacterium pseudolongum* in mediating the beneficial effects of OFS on nutrient-induced satiation. These findings highlight the importance of the small intestinal microbiota and small intestinal nutrient sensing in metabolic homeostasis and establish the small intestinal microbiota as a potential target for treatment of obesity via regulation of food intake.

## Supplementary Information

**Additional file 1.**

## Data Availability

All data generated or analyzed during this study are included in this published article and its supplementary information files.
