# Supplementary material for: Oligofructose improves small intestinal lipid-sensing mechanisms via alterations to the small intestinal microbiota
Source: Microbiome. 2023 Aug 2;11:169. doi: 10.1186/s40168-023-01590-2 (PMC10394784; doi:10.1186/s40168-023-01590-2)
Supplement: Supplementary file 2 — Additional file 1. [file 40168_2023_1590_MOESM1_ESM.zip › 1-Additional File 1.pdf]

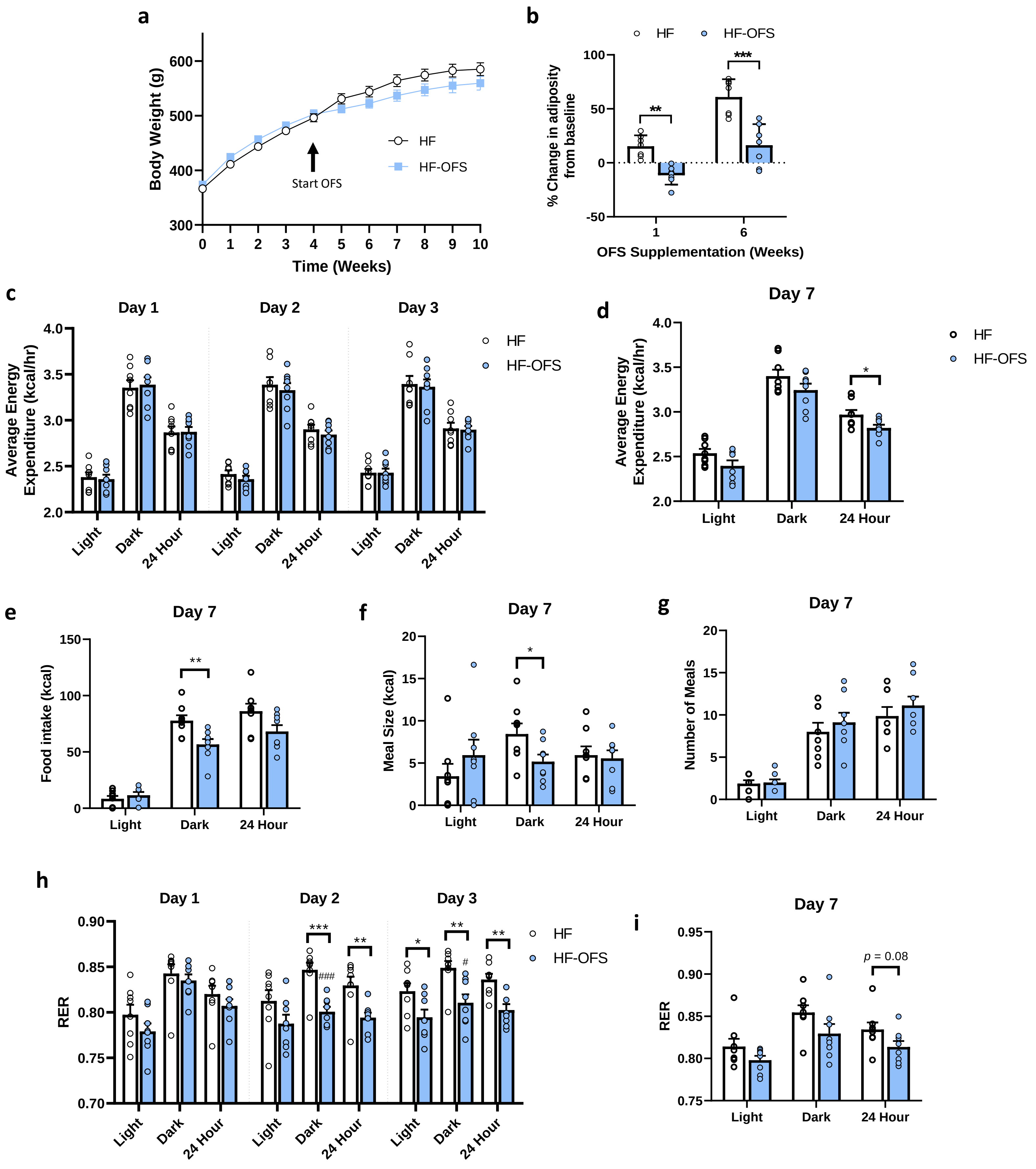

Supplementary Figure 1. Food intake and energy metabolism following acute OFS treatment. (a) 10-week bodyweight and (b) percent change in adiposity at 1- and 6-weeks following OFS treatment. Average energy expenditure in (c) the first 3 days and (d) after 7 days of OFS treatment. (e) Food intake, (f) meal size, and (g) number of meals after 7 days of OFS treatment. RER in (h) the first 3 days and (i) after 7 days of OFS treatment. Data in all graphs represent the mean + SEM (n=8 per group); $*p < 0.05$, $**p < 0.01$, $***p < 0.001$ vs HF-OFS; ; $\#p < 0.05$, $\#\#p < 0.01$, $\#\#\#p < 0.001$ vs day, as assessed by two-way ANOVA with Šídák's multiple comparisons test or Welch's *t* test.

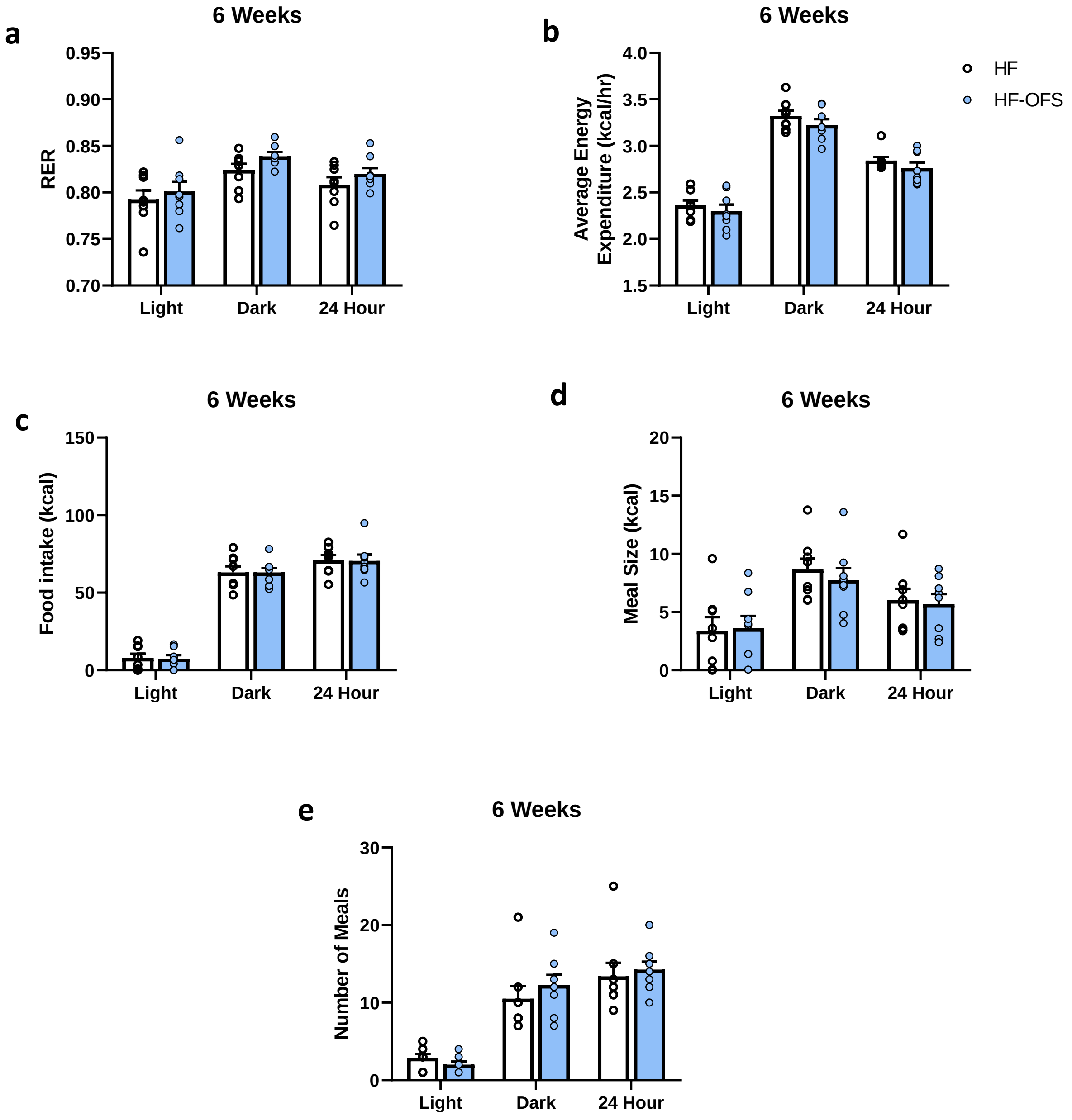

Supplementary Figure 2. Food intake and energy metabolism following 6-week OFS treatment. (a) RER, (b) EE, (c) food intake, (d) meal size, and (e) number of meals following 6 weeks of OFS treatment or maintenance on a HF diet. Data in all graphs represent the mean + SEM (n=8 per group); *p < 0.05, **p < 0.01, ***p < 0.001 vs HF-OFS; ; #p < 0.05, ##p < 0.01, ###p < 0.001 vs day, as assessed by two-way ANOVA with Šídák's multiple comparisons test or Welch's *t* test.

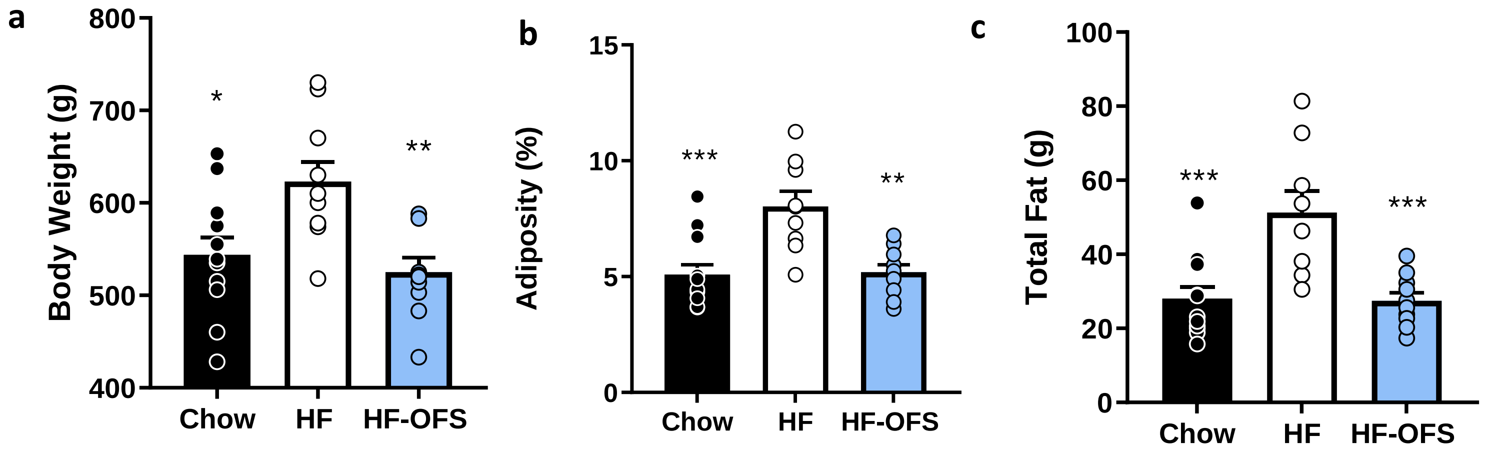

Supplementary Figure 3. OFS supplementation reduces bodyweight and adiposity in rats on a HF diet. Changes in (a) bodyweight, (b) adiposity, and (c) total fat mass following 6-weeks of OFS supplementation in drinking water. Data in all graphs represent the mean + SEM (n=6-8 per group); *$p < 0.05$, **$p < 0.01$, ***$p < 0.001$ vs HF as assessed by one-way ANOVA.

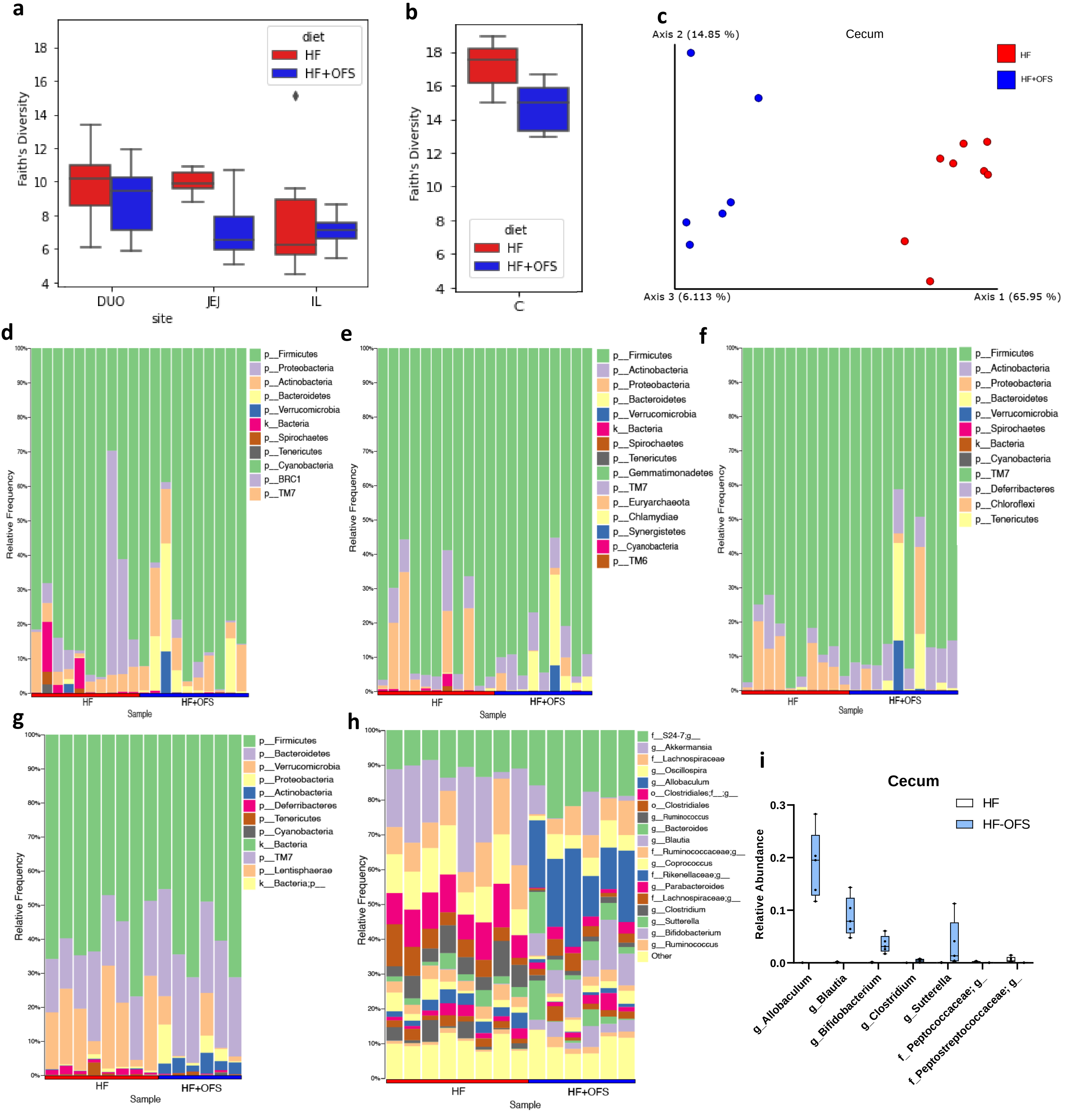

Supplementary Figure 4. Long-term OFS treatment beneficially alters the gut microbiota. Alpha diversity in (a) each site of the small intestine and (b) cecum. Principal coordinate analysis (PCoA) of weighted UniFrac distances of the (c) cecal microbial profiles between HF (red) and HF-OFS (blue) rats. Axis indicate the percentage of variation explained by the plotted principal coordinates. Phylum level analysis of the relative abundance in the (d) duodenum, (e) jejunum, (f) ileum, and (g) cecum of HF and HF-OFS rats. Genus level analysis of the relative abundance in the (h) cecum of HF and HF-OFS rats. Box and whisker plots of the relative abundance of bacterial genus with significantly altered abundance between HF and HF-OFS rats in the (i) cecum.

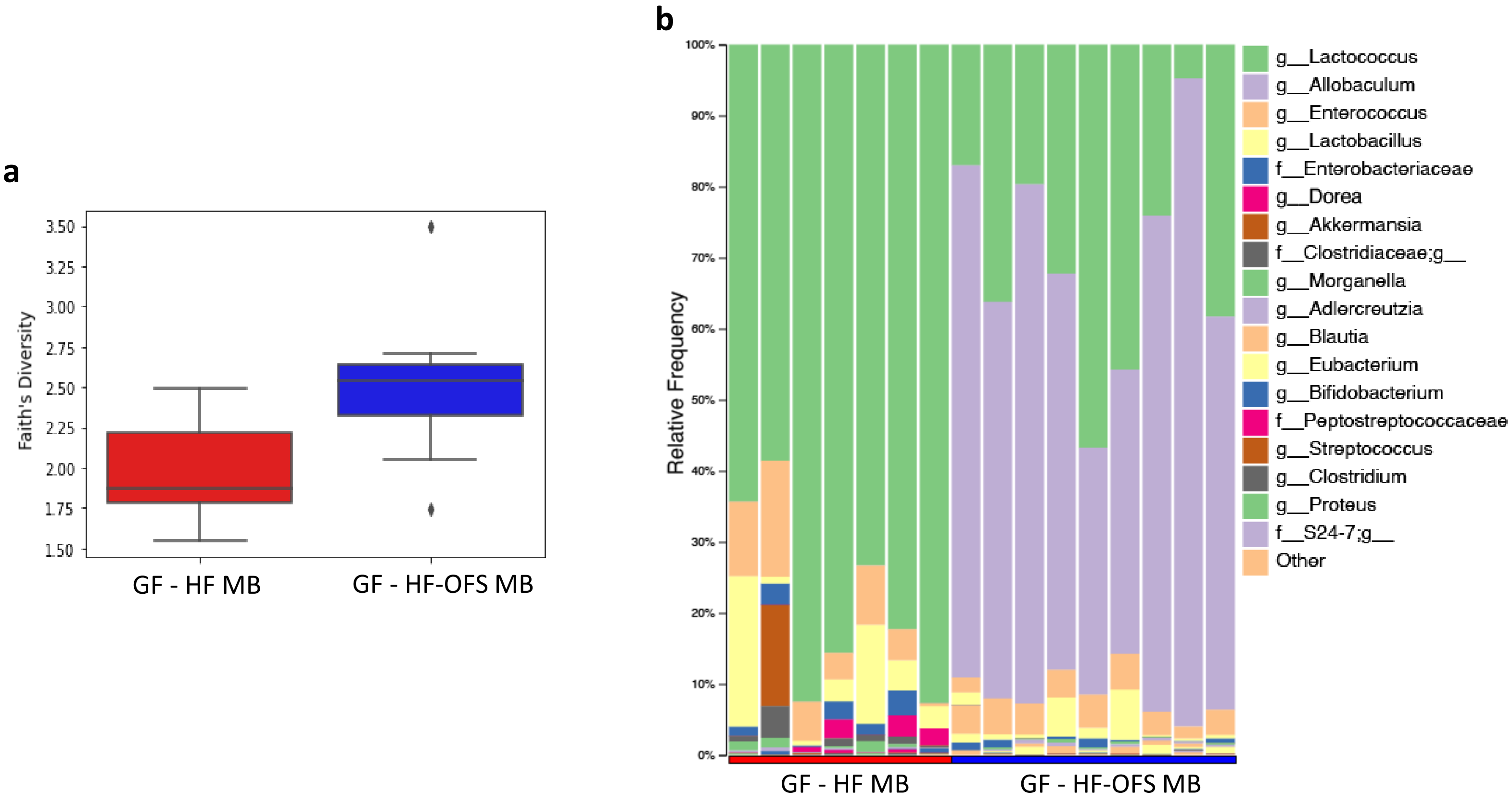

Supplementary Figure 5. Shifts in the SI microbiota of germ-free mice following inoculation with the SI microbiota of HF or HF-OFS rats. (a) Alpha diversity and (b) taxonomic analysis of the relative frequency of bacterial genera in the small intestine of HF-fed, germ-free mice 3 weeks following inoculation with the SI microbiota of HF (n=7) or HF-OFS rats (n=9).

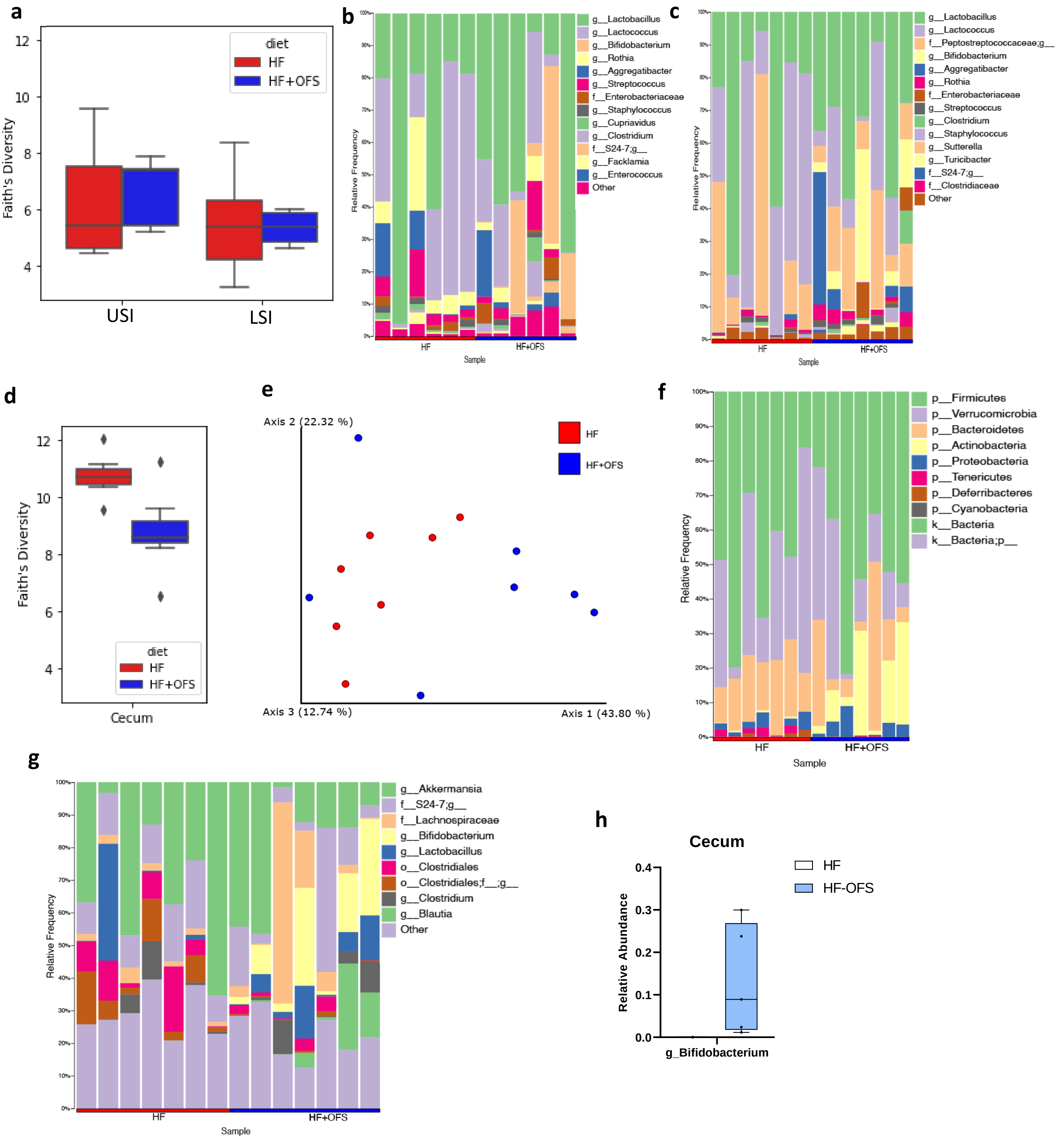

Supplementary Figure 6. Acute OFS treatment beneficially alters the gut microbiota. Alpha diversity in (a) each site of the small intestine and (d) cecum. Genus level analysis of the relative abundance in the (b) USI and (c) LSI of HF and HF-OFS rats. Weighted UniFrac distances of the (e) cecal microbial profile between HF (red) and HF-OFS (blue) rats. Axis indicate the percentage of variation explained by the plotted principal coordinates. Phylum level analysis of the relative abundance in the (f) cecum of HF and HF-OFS rats. Genus level analysis of the relative abundance in the (g) cecum of HF and HF-OFS rats. Box and whisker plots of the relative abundance of bacterial genus with significantly altered abundance between HF and HF-OFS rats in the (h) cecum**.**

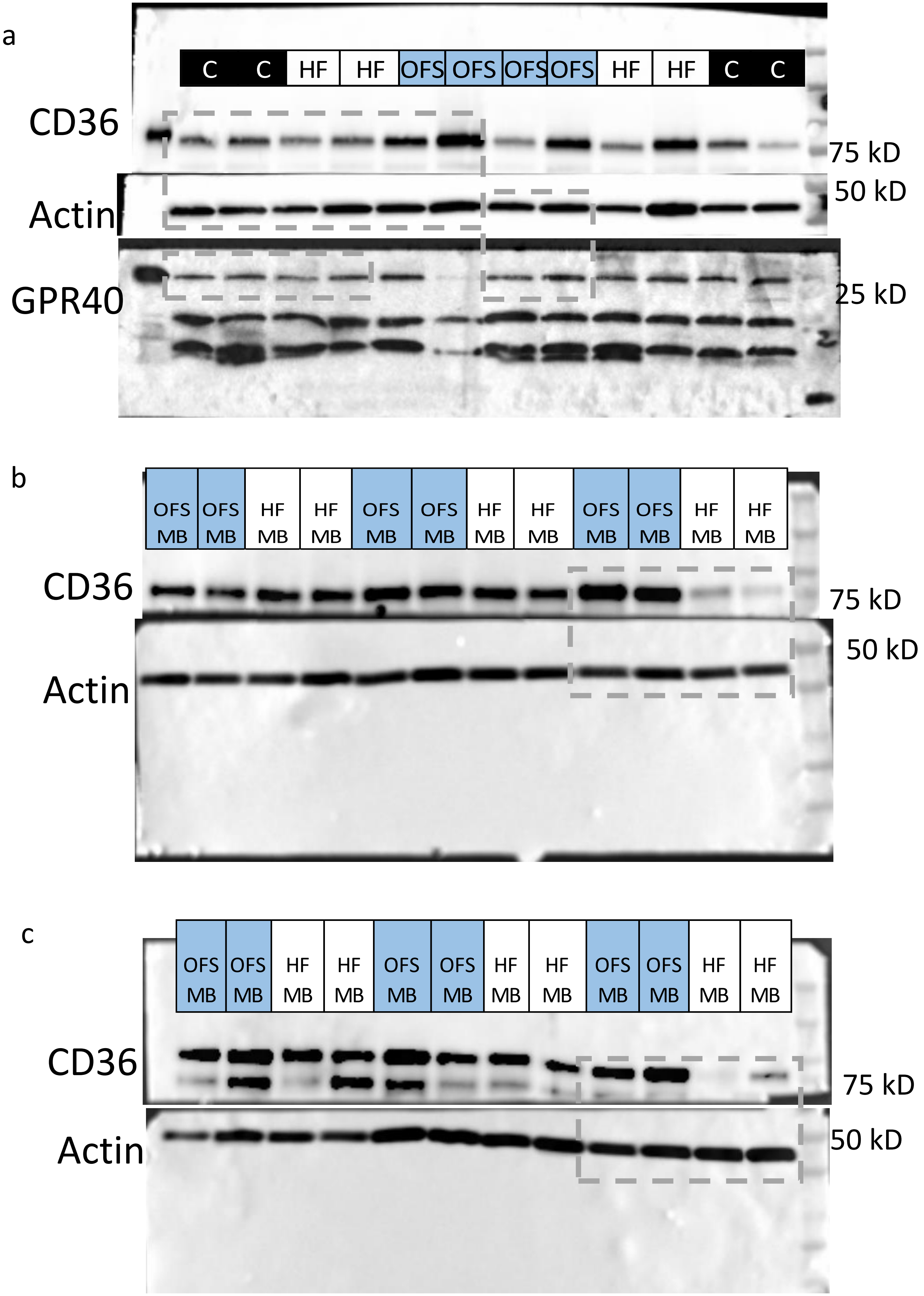

Supplementary Figure 7. Uncropped western blot of the (a) jejunum of 6-week OFS treated rats and the (b) USI and (c) LSI following microbiota transplant from HF-OFS rats to HF rats and from HF rats to HF-OFS rats. Grey boxes indicate representative images used in manuscript.

Supplementary Table 1. Calorie, macronutrient, and micronutrient composition for chow, HF and HF-OFS diets.

	HF Diet (Research Diets D12451)	HF- OFS Diet (Research Diets D19112708)		Chow Diet (Teklad 2018)
**Macronutrients**	***kcal%***	***kcal%***		***kcal%***
Protein	*20.0*	*20.0*		*24.0*
Carbohydrate	*35.0*	*35.0*		*58.0*
Fat	*45.0*	*45.0*		*18.0*
**kcal/gm**	**4.73**	**4.71**		**3.10**
**Micronutrients**				
**Vitamin Mix V10001**				
Vit A	*4800 IU/kg*	*4800 IU/kg*		*15000 IU/kg*
Vit D3	*1200 IU/kg*	*1200 IU/kg*		*1500 IU/kg*
Vit E	*60 IU/ kg*	*61 IU/ kg*		*110 IU/kg*
Menadione	*0.6 mg/kg*	*0.6 mg/kg*		*50 mg/kg*
Biotin	*0.24 mg/kg*	*0.24 mg/kg*		*0.4 mg/kg*
Vit B12	*12 μg/kg*	*13 μg/kg*		*80 mg/kg*
Folic Acid	*2.4 mg/kg*	*2.4 mg/kg*		*0.4 mg/kg*
Niacin	*36 mg/kg*	*37 mg/kg*		*70 mg/kg*
Pantothenic Acid	*19.2 mg/kg*	*19.2 mg/kg*		*33 mg/kg*
Vit B6	*8.4 mg/kg*	*8.4 mg/kg*		*18 mg/kg*
Vit B2	*7.2 mg/kg*	*7.2 mg/kg*		*15 mg/kg*
Vit B1	*7.2 mg/kg*	*7.2 mg/kg*		*17 mg/kg*
Choline Bitartrate	*2.3 g/kg*	*2.3 g/kg*		*1.2 g/kg*
**Mineral Mix S10026**				
Sodium	*1.2 g/ kg*	*1.2 g/ kg*		*0.2%*
Chloride	*1.92 g/ kg*	*1.92 g/ kg*		*0.4%*
Magnesium	*0.6 g/ kg*	*0.6 g/ kg*		*0.2%*
Magnesium Sulfate	*0.396 g/ kg*	*0.396 g/ kg*		
Ammonium Molybdate	*1.92 mg/kg*	*1.92 mg/kg*		
Chromium Potassium Sulfate	*2.4 mg/kg*	*2.4 mg/kg*		
Copper	*7.2 mg/kg*	*7.2 mg/kg*		*15 mg/kg*
Iron	*44.4 mg/kg*	*44.4 mg/kg*		*200 mg/kg*
Manganese	*70.8 mg/kg*	*70.8 mg/kg*		*100 mg/kg*
Iodine	*0.24 mg/kg*	*0.24 mg/kg*		*6 mg/kg*
Flouride	*1.08 mg/kg*	*1.08 mg/kg*		
Selenium	*0.192 mg/kg*	*0.192 mg/kg*		*0.23 mg/kg*
Zinc	*34.8 mg/kg*	*34.8 mg/kg*		*70 mg/kg*
Dicalcium Phosphate	*15.1 g/kg*	*15.1 g/kg*		*1.0%*
Calcium Carbonate	*6.4 g/kg*	*6.4 g/kg*		*0.7%*
Potassium Citrate, 1 H2O	*19.2g/kg*	*19.2g/kg*		*0.6%*

Supplementary Table 2. Correlation analysis of significantly altered bacterial geneses with bodyweight and adiposity in 6-week OFS-treated rats.

	Bodweight		Adiposity	
**Duodendum**	**R**	***p* value**	**R**	***p* value**
k__Bacteria;p__Firmicutes;c__Erysipelotrichi;o__Erysipelotrichales;f__Erysipelotrichaceae;g__Allobaculum	-0.5443	0.0160	-0.6134	0.0052
k__Bacteria;p__Firmicutes;c__Clostridia;o__Clostridiales;f__Lachnospiraceae;g__Blautia	-0.5389	0.0173	-0.5715	0.0106
k__Bacteria;p__Firmicutes;c__Bacilli;o__Bacillales;f__Staphylococcaceae;g__Staphylococcus	0.4707	0.0420	0.5775	0.0096
**Jejunum**				
k__Bacteria;p__Firmicutes;c__Erysipelotrichi;o__Erysipelotrichales;f__Erysipelotrichaceae;g__Allobaculum	-0.6968	0.0009	-0.7667	0.0001
k__Bacteria;p__Bacteroidetes;c__Bacteroidia;o__Bacteroidales;f__S24-7;g__	-0.8165	2.00E-05	-0.7372	0.0003
k__Bacteria;p__Firmicutes;c__Clostridia;o__Clostridiales;f__Lachnospiraceae;g__Blautia	-0.5303	0.0195	-0.5186	0.0229
k__Bacteria;p__Firmicutes;c__Clostridia;o__Clostridiales;f__Peptostreptococcaceae;__	0.5717	0.0105	0.4584	0.0484
**Ileum**				
k__Bacteria;p__Firmicutes;c__Erysipelotrichi;o__Erysipelotrichales;f__Erysipelotrichaceae;g__Allobaculum	-0.5360	0.0180	-0.6248	0.0042
k__Bacteria;p__Actinobacteria;c__Actinobacteria;o__Bifidobacteriales;f__Bifidobacteriaceae;g__Bifidobacterium	-0.6910	0.0011	-0.7223	0.0005
k__Bacteria;p__Firmicutes;c__Clostridia;o__Clostridiales;f__Peptostreptococcaceae;__	0.7182	0.0005	0.6099	0.0056
k__Bacteria;p__Firmicutes;c__Clostridia;o__Clostridiales;f__Lachnospiraceae;g__Blautia	-0.6369	0.0034	-0.8066	3.02E-05
**Cecum**				
k__Bacteria;p__Firmicutes;c__Erysipelotrichi;o__Erysipelotrichales;f__Erysipelotrichaceae;g__Allobaculum	-0.4472	0.1089	-0.6245	0.0170
k__Bacteria;p__Actinobacteria;c__Actinobacteria;o__Bifidobacteriales;f__Bifidobacteriaceae;g__Bifidobacterium	-0.4683	0.0912	-0.4645	0.0943
k__Bacteria;p__Firmicutes;c__Clostridia;o__Clostridiales;f__Lachnospiraceae;g__Blautia	-0.5569	0.0386	-0.5188	0.0573
k__Bacteria;p__Firmicutes;c__Clostridia;o__Clostridiales;f__Lachnospiraceae;g__Clostridium	-0.6993	0.0054	-0.6328	0.0151
k__Bacteria;p__Proteobacteria;c__Betaproteobacteria;o__Burkholderiales;f__Alcaligenaceae;g__Sutterella	-0.8800	3.38E-05	-0.7219	0.0036
k__Bacteria;p__Firmicutes;c__Clostridia;o__Clostridiales;f__Peptococcaceae;g__	0.6188	0.0183	0.5237	0.0546
k__Bacteria;p__Firmicutes;c__Clostridia;o__Clostridiales;f__Peptostreptococcaceae;__	0.6051	0.0218	0.4689	0.0908
