# Supplementary material for: Oligofructose improves small intestinal lipid-sensing mechanisms via alterations to the small intestinal microbiota
Source: Microbiome. 2023 Aug 2;11:169. doi: 10.1186/s40168-023-01590-2 (PMC10394784; doi:10.1186/s40168-023-01590-2)
Supplement: Supplementary file 2 — Additional file 1. [file 40168_2023_1590_MOESM1_ESM.zip › 2-Additional File 1.pdf]

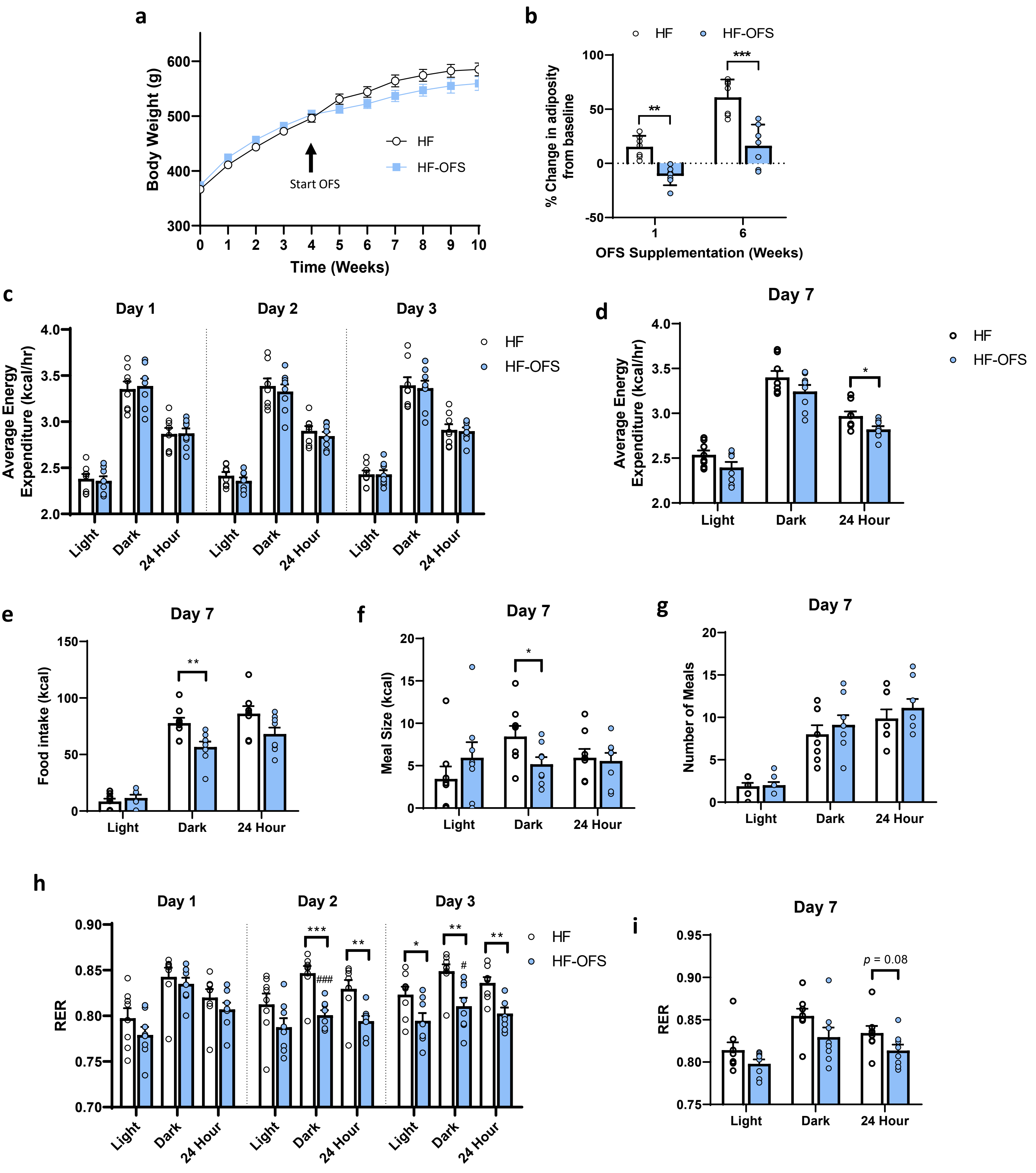

Supplementary Figure 1. Food intake and energy metabolism following acute OFS treatment. (a) 10-week bodyweight and (b) percent change in adiposity at 1- and 6-weeks following OFS treatment. Average energy expenditure in (c) the first 3 days and (d) after 7 days of OFS treatment. (e) Food intake, (f) meal size, and (g) number of meals after 7 days of OFS treatment. RER in (h) the first 3 days and (i) after 7 days of OFS treatment. Data in all graphs represent the mean + SEM (n=8 per group); *p < 0.05, **p < 0.01, ***p < 0.001 vs HF-OFS; ; #p < 0.05, ##p < 0.01, ###p < 0.001 vs day, as assessed by two-way ANOVA with Šídák's multiple comparisons test or Welch's *t* test.

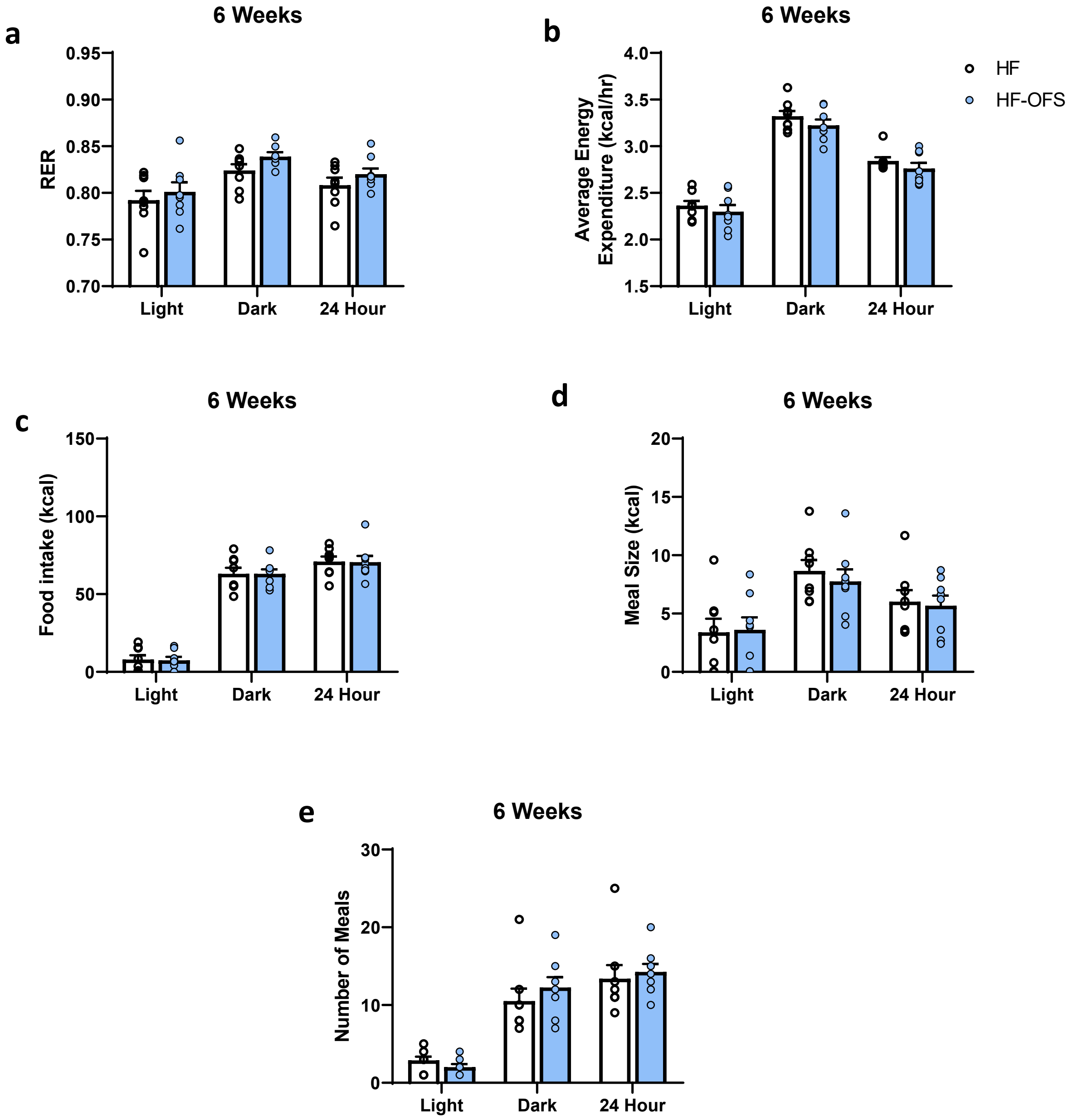

Supplementary Figure 2. Food intake and energy metabolism following 6-week OFS treatment. (a) RER, (b) EE, (c) food intake, (d) meal size, and (e) number of meals following 6 weeks of OFS treatment or maintenance on a HF diet. Data in all graphs represent the mean + SEM (n=8 per group); *$p < 0.05$, **$p < 0.01$, ***$p < 0.001$ vs HF-OFS; ; #$p < 0.05$, ##$p < 0.01$, ###$p < 0.001$ vs day, as assessed by two-way ANOVA with Šídák's multiple comparisons test or Welch's *t* test.

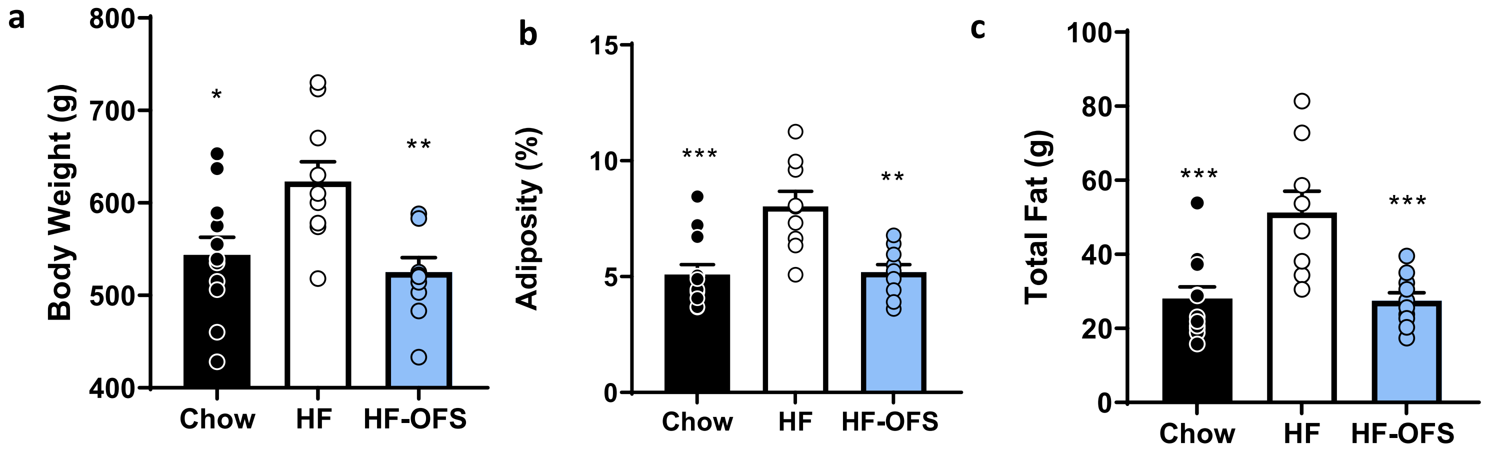

Supplementary Figure 3. OFS supplementation reduces bodyweight and adiposity in rats on a HF diet. Changes in (a) bodyweight, (b) adiposity, and (c) total fat mass following 6-weeks of OFS supplementation in drinking water. Data in all graphs represent the mean + SEM (n=6-8 per group); *$p < 0.05$, **$p < 0.01$, ***$p < 0.001$ vs HF as assessed by one-way ANOVA.

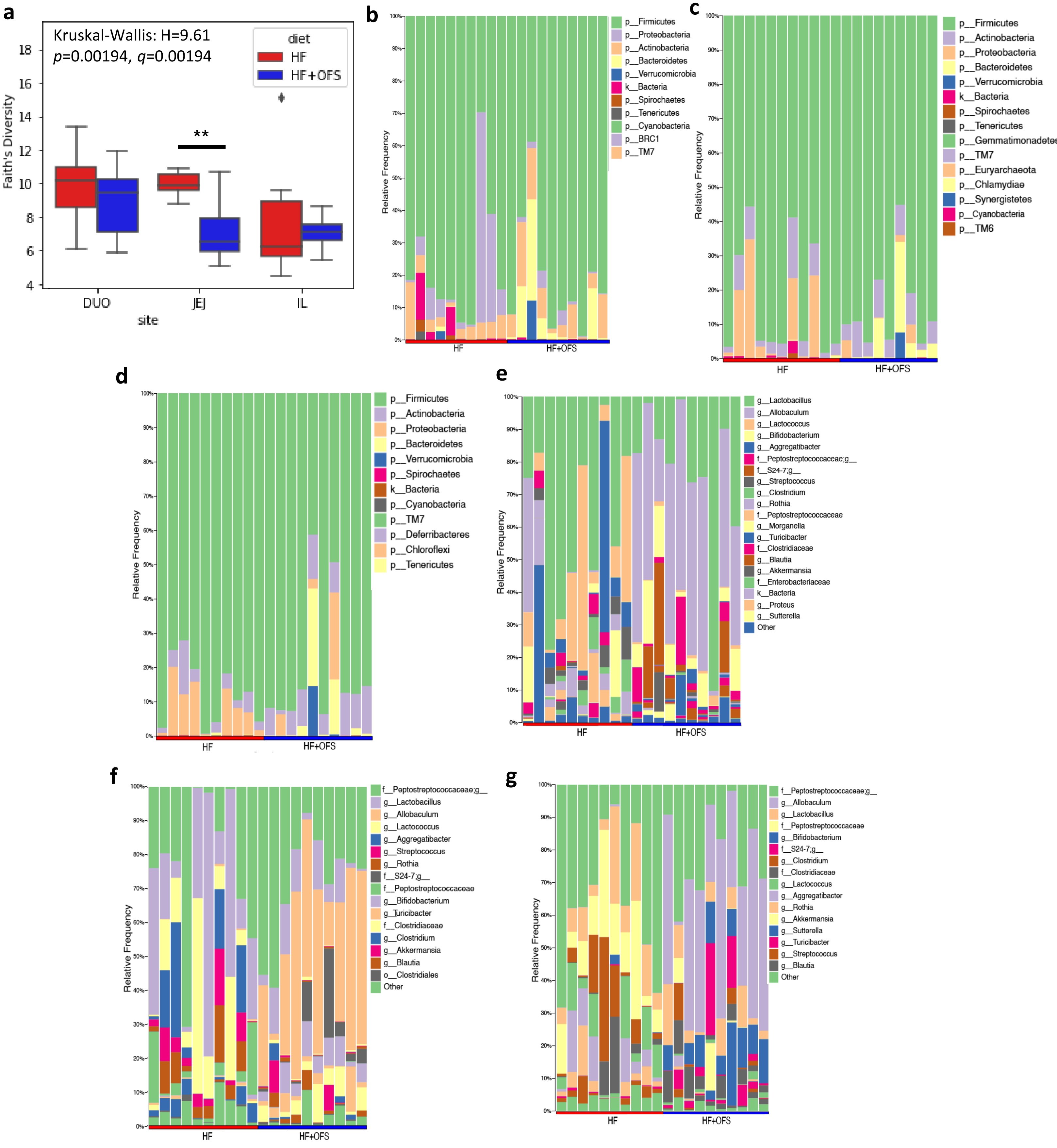

Supplementary Figure 4. Long-term OFS treatment beneficially alters the small intestinal gut microbiota. Alpha diversity in (a) each site of the small intestine. Phylum level analysis of the relative frequency in the (b) duodenum, (c) jejunum, and (d) ileum of HF and HF-OFS rats. Genus level analysis of the relative frequency in the (e) duodenum, (f) jejunum, and (g) ileum of HF and HF-OFS rats.

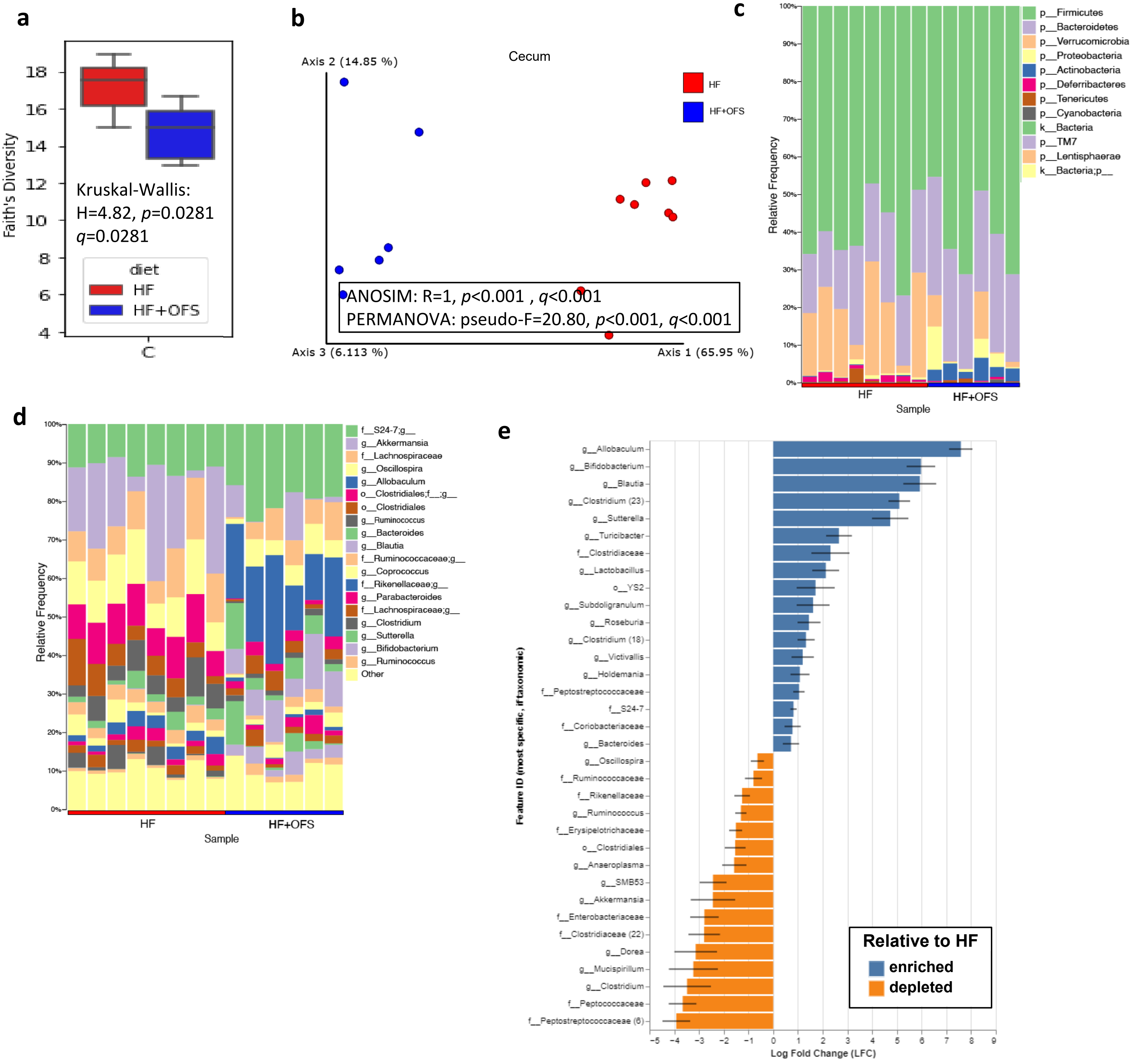

Supplementary Figure 5. Long-term OFS treatment beneficially alters the cecal microbiota. (a) Alpha diversity in the cecum. (b) Principal coordinate analysis (PCoA) of weighted UniFrac distances of the cecal microbial profiles between HF (red) and HF-OFS (blue) rats. Axis indicate the percentage of variation explained by the plotted principal coordinates. (c) Phylum and (d) genus level analysis of the relative abundance in the cecum of HF and HF-OFS rats. (e) Log-Fold Change of bacterial genera in HF-OFS rats compared to HF rats in the cecum. Different taxonomic annotation with the same genus label are appended with numbers in order to separate the values. The feature labels (y-axis labels) in each plot represent the most specific named taxonomic level describing the feature. Feature identifiers that are duplicated represent instances of a duplicated taxonomic name at the taxonomic level displayed in the feature identifier. The number following the feature identifiers in these cases is used only for unique identification in the current figure. It is not taxonomically meaningful.

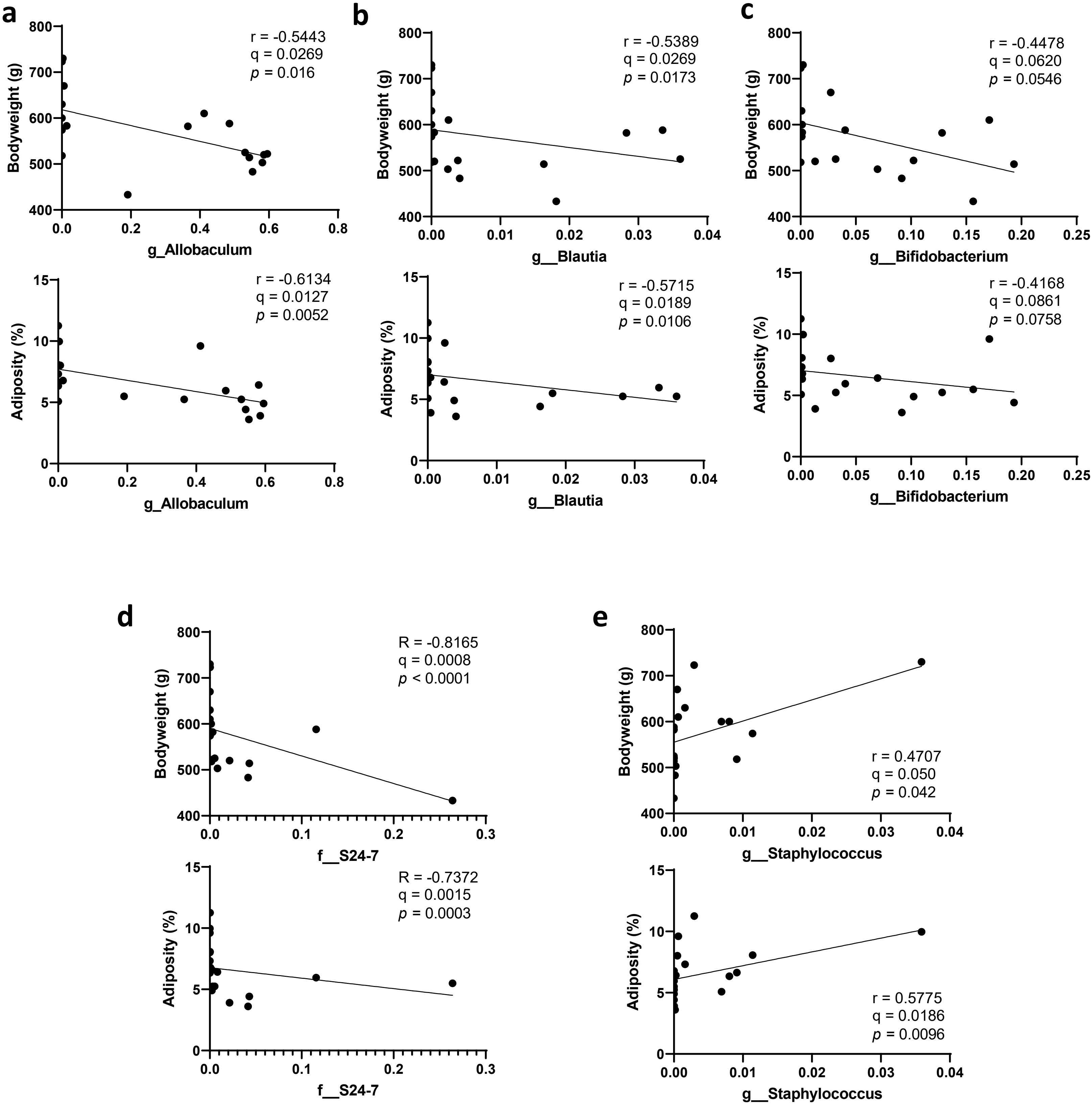

Supplementary Figure 6. Duodenum correlation analysis. Correlation analysis of body weight and adiposity with bacterial families and genera with a log fold change > 4 (A-C) or < -4 (D) in the duodenum from HF or 6-week HF-OFS-treated rats.

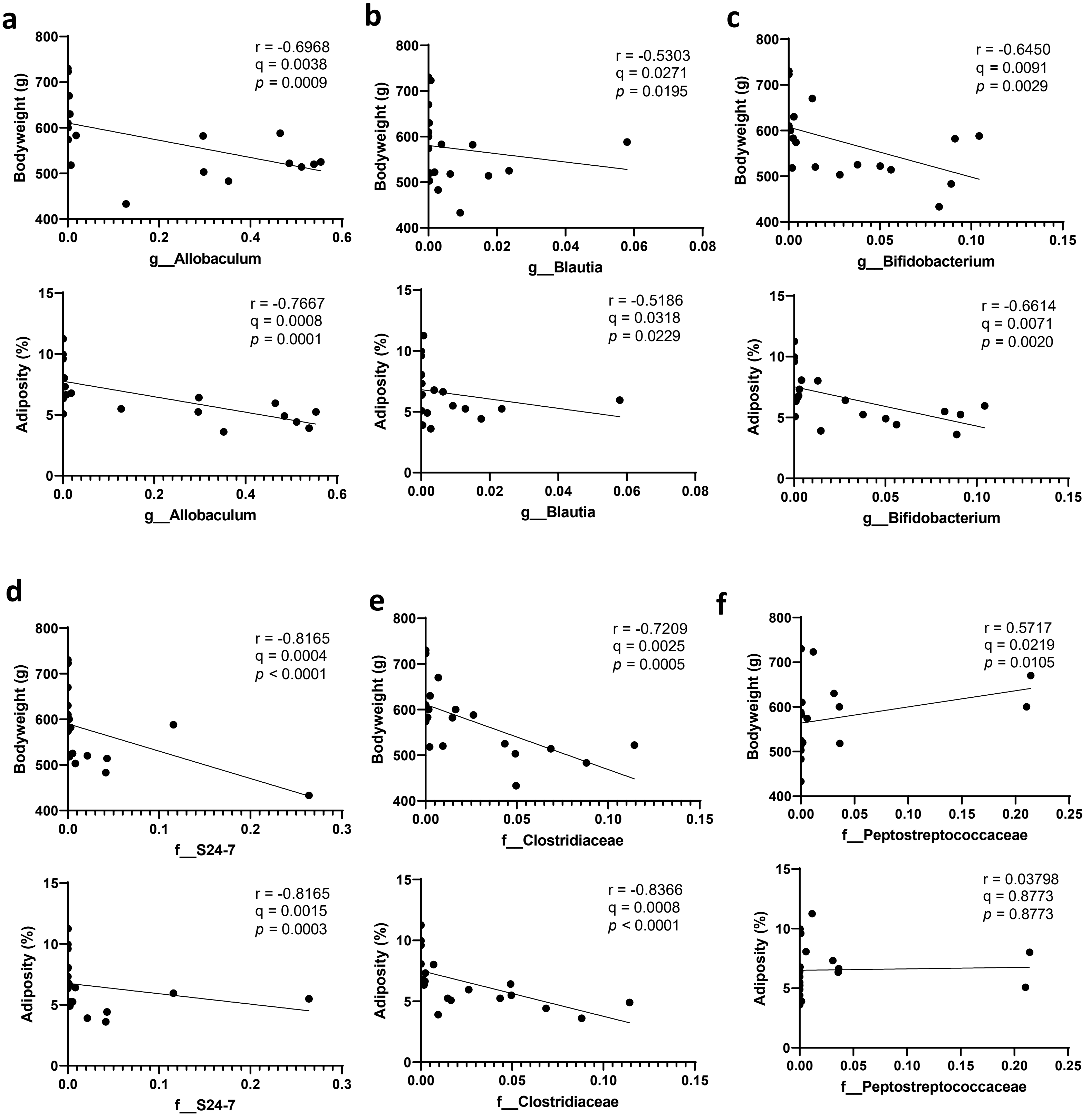

Supplementary Figure 7. Jejunum correlation analysis. Correlation analysis of body weight and adiposity with bacterial families and genera with a log fold change > 4 (A-E) or < -4 (F) in the jejunum from HF or 6-week HF-OFS-treated rats.

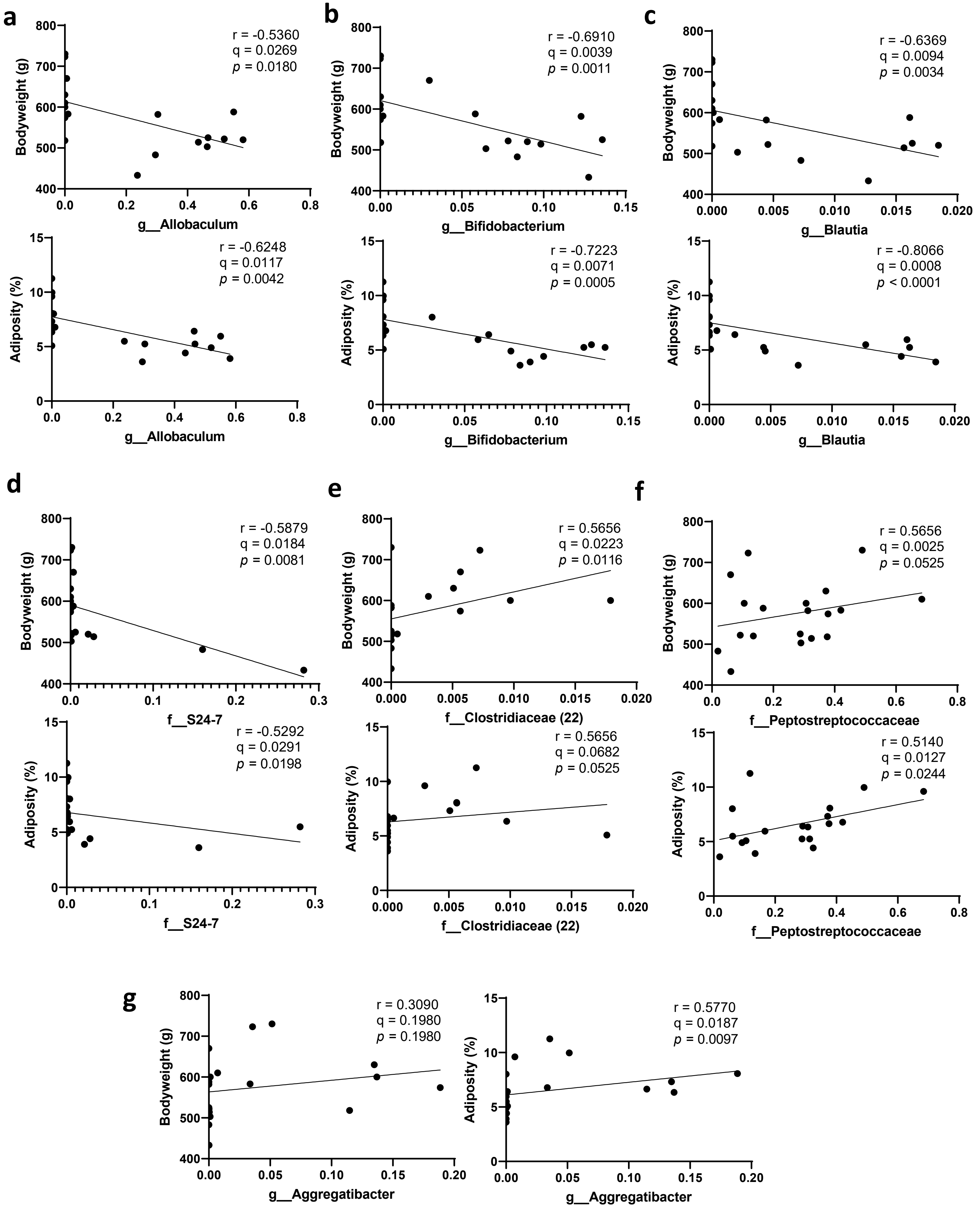

Supplementary Figure 8. Ileum correlation analysis. Correlation analysis of body weight and adiposity with bacterial families and genera with a log fold change > 4 (A-D) or < -4 (F-G) in the ileum from HF or 6-week HF-OFS-treated rats.

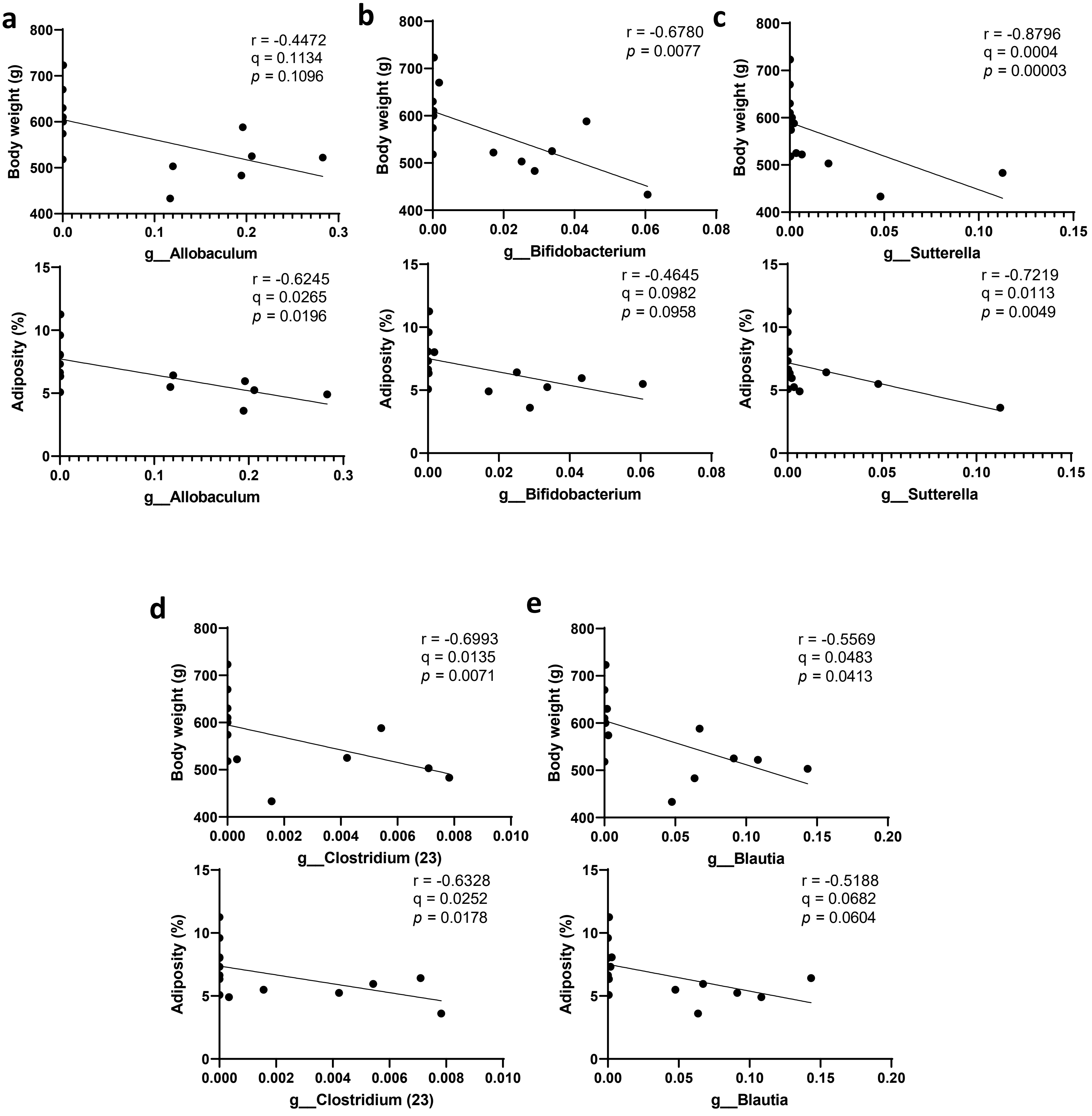

Supplementary Figure 9. Cecal correlation analysis. Correlation analysis of body weight and adiposity with bacterial families and genera with a log fold change > 4 (A-E) in the cecum from HF or 6-week HF-OFS-treated rats.

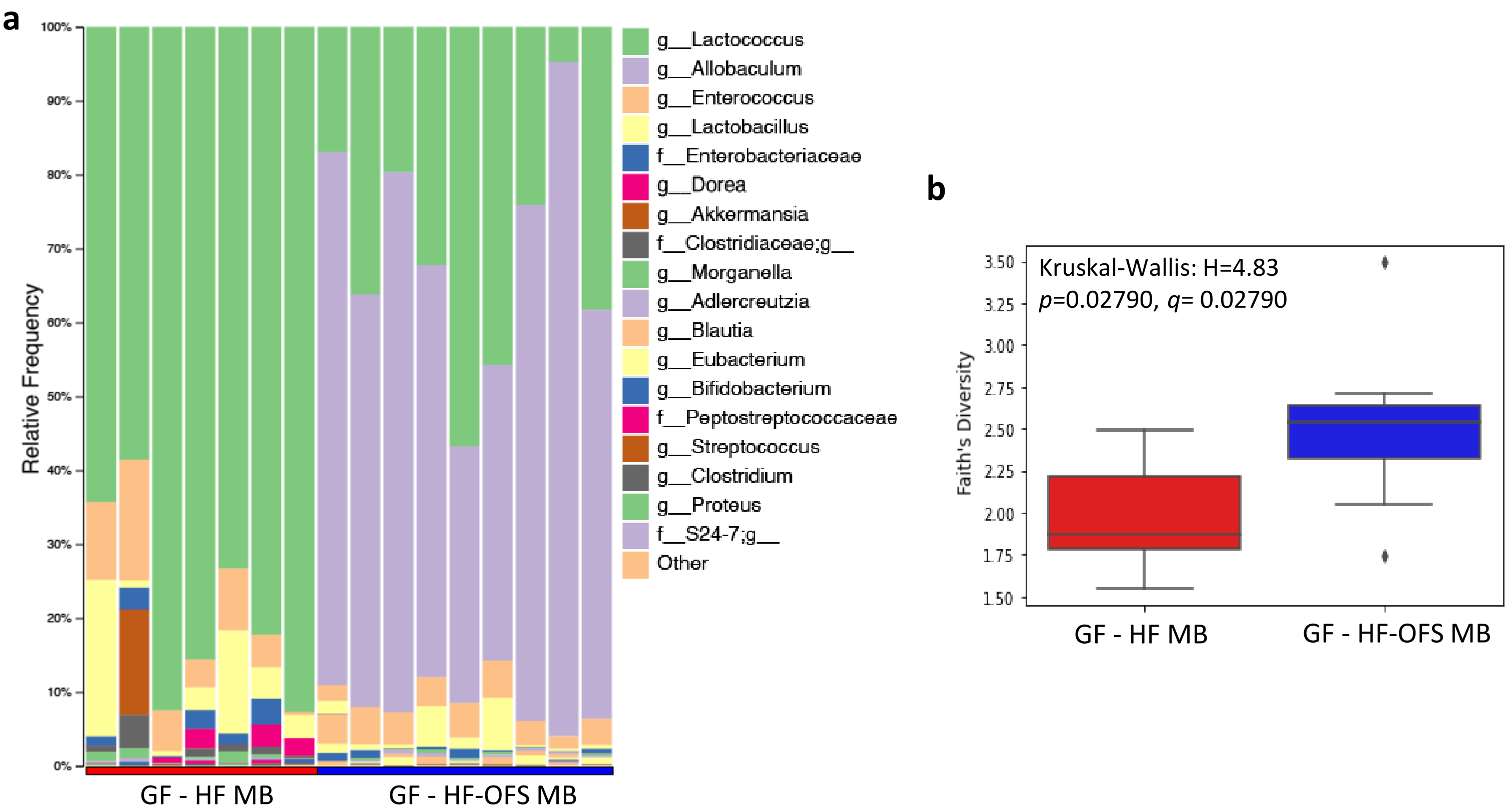

Supplementary Figure 10. Shifts in the SI microbiota of germ-free mice following inoculation with the SI microbiota of HF or HF-OFS rats. (a) Taxonomic analysis (b) alpha diversity of the relative frequency of bacterial genera in the small intestine of HF-fed, germ-free mice 3 weeks following inoculation with the SI microbiota of HF (n=7) or HF-OFS rats (n=9).

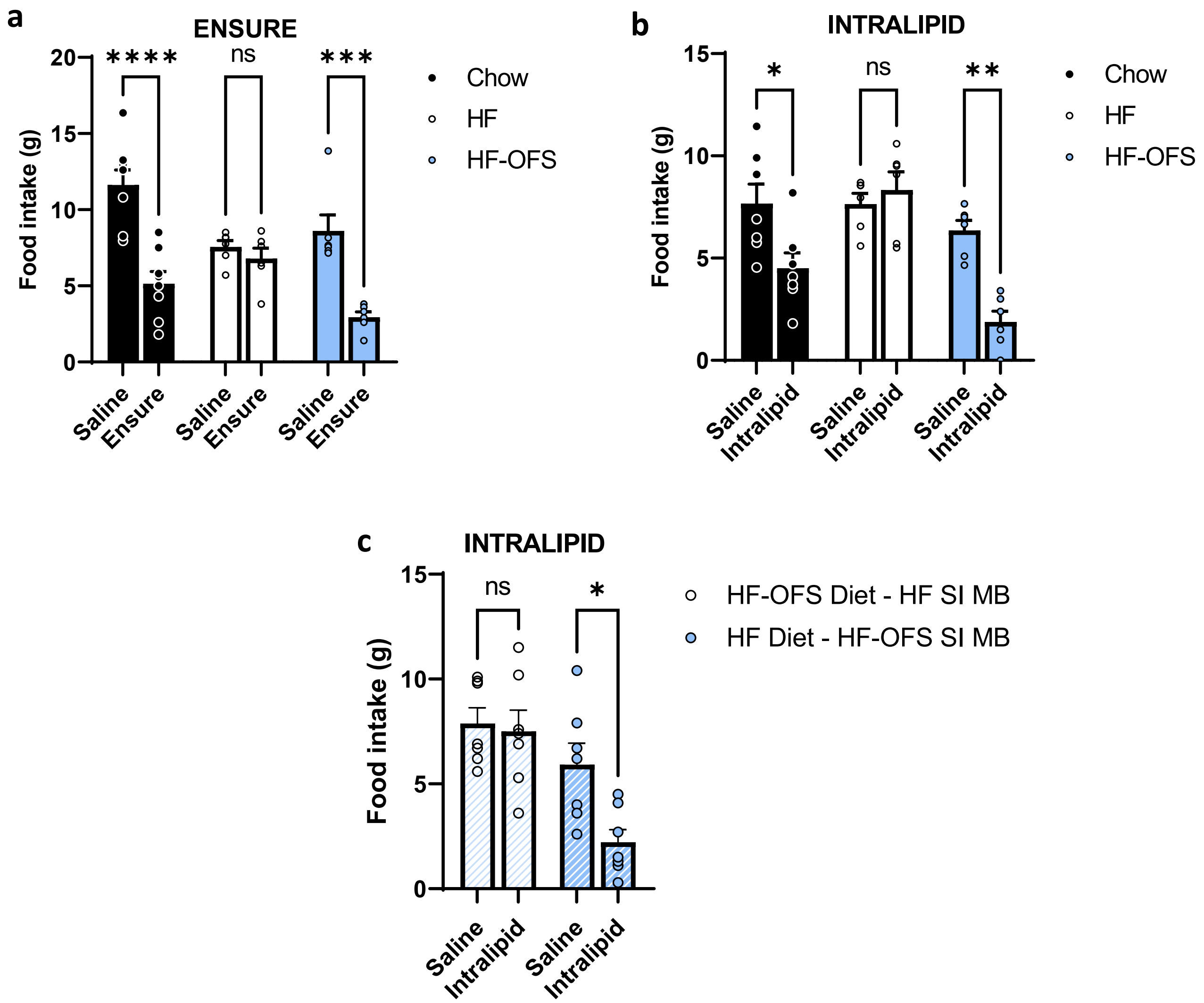

Supplementary Figure 11. Nutrient-induced satiation food intake values. (a) Food intake following saline or Ensure SI infusion in rats on a chow diet, HF diet, or HF diet with OFS supplementation. (b) Food intake following saline or Intralipid SI infusion in rats on a chow diet, HF diet, or HF diet with OFS supplementation. (c) Food intake following saline or Intralipid SI infusion in rats following a SI microbiota transplant. Data in all graphs represent the mean + SEM (n=6-7 per group); *$p < 0.05$, **$p < 0.01$, ***$p < 0.001$ vs HF as assessed by two-way ANOVA.

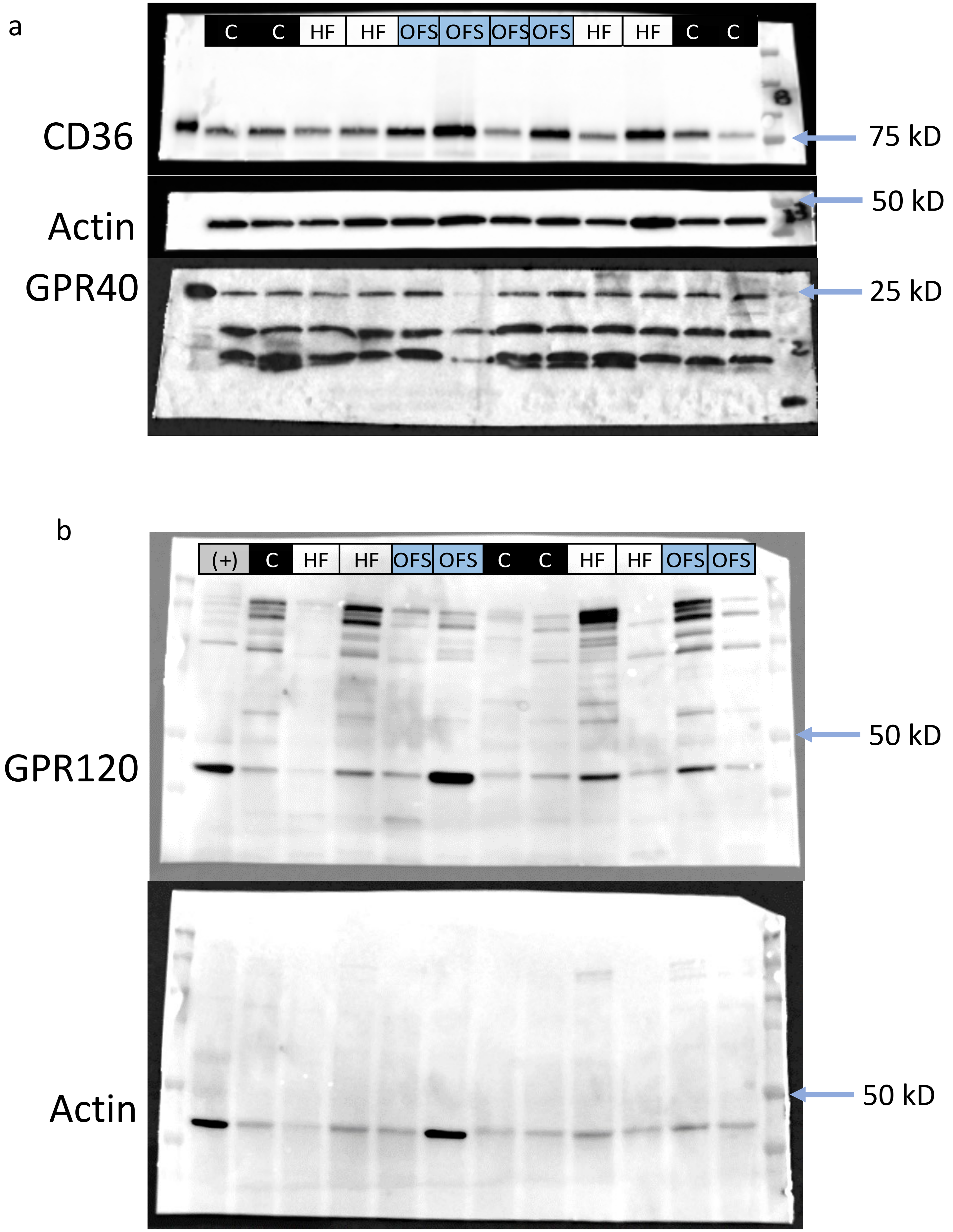

Supplementary Figure 12. Uncropped western blot of (a) CD36, GPR40, and (b) GPR120 from the jejunum of 6-week OFS treated rats. Lung was used as a positive control for GPR120.

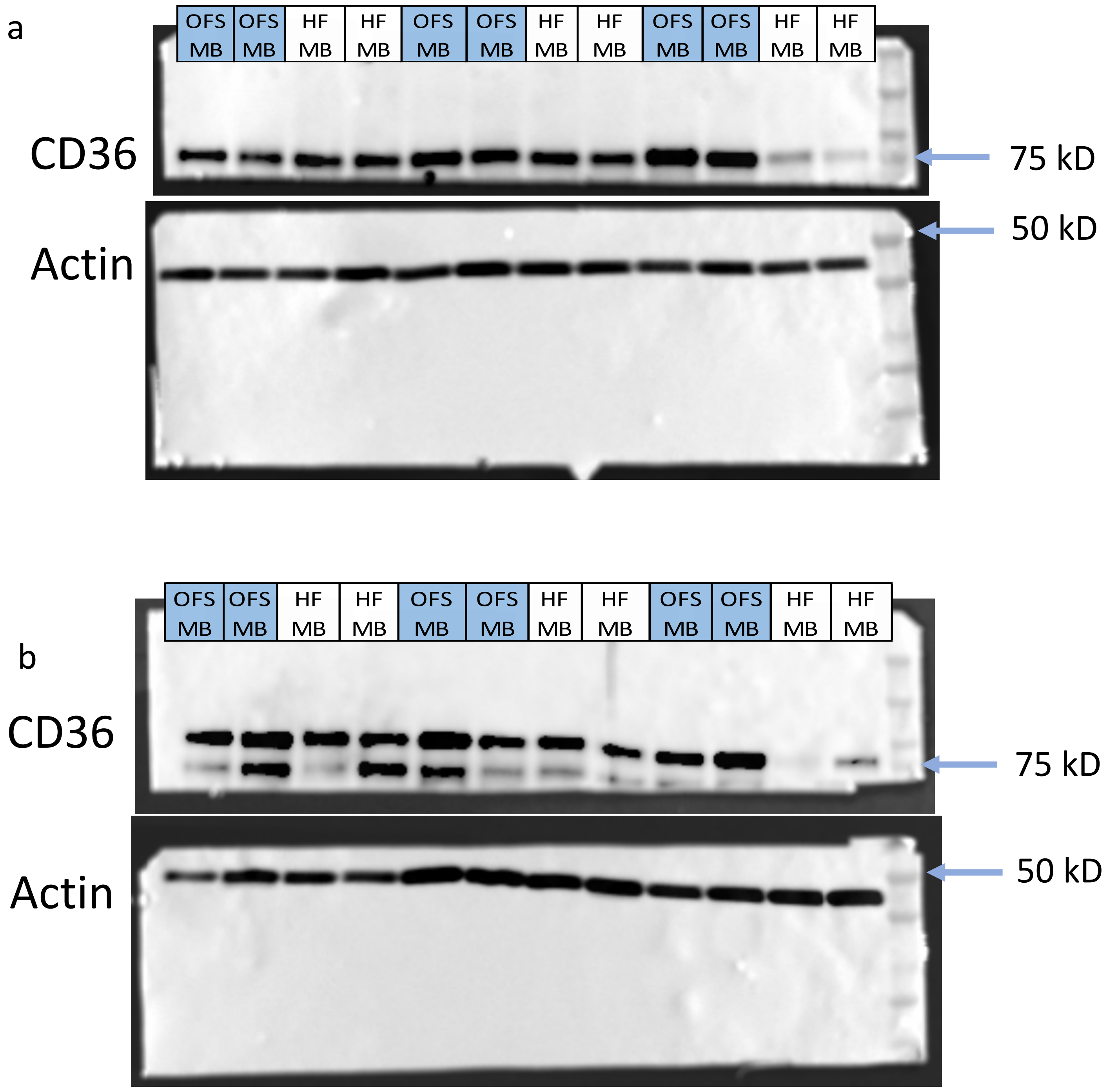

Supplementary Figure 13. Uncropped western blot of the (a) USI and (b) LSI following microbiota transplant from HF-OFS rats to HF rats and from HF rats to HF-OFS rats. Membranes were cut and incubated in respective primary antibodies to prevent the need to strip and reprobe for multiple proteins.

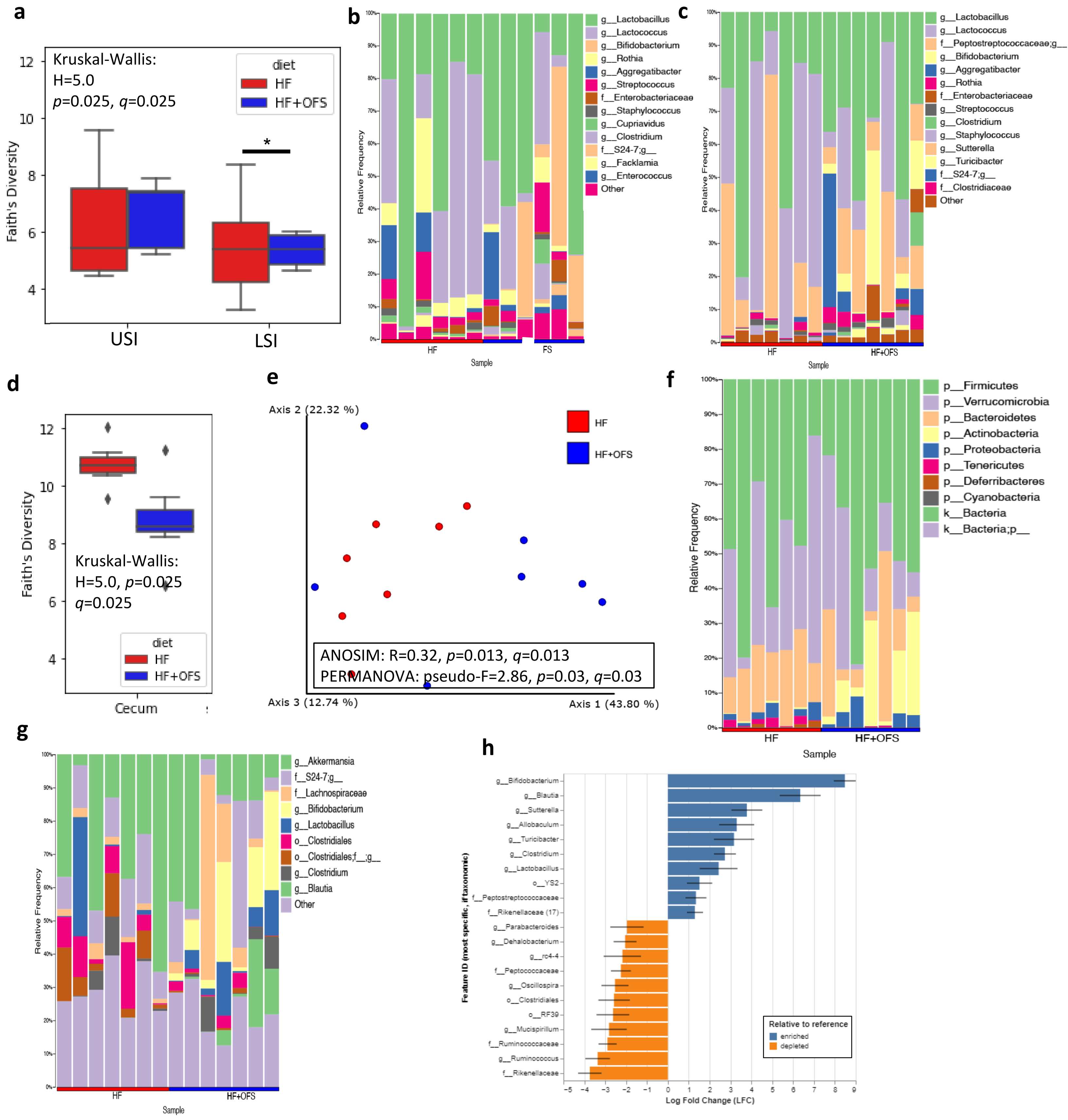

Supplementary Figure 14. Acute OFS treatment beneficially alters the gut microbiota. Alpha diversity in (a) each site of the small intestine and (d) cecum. Genus level analysis of the relative abundance in the (b) USI and (c) LSI of HF and HF-OFS rats. Weighted UniFrac distances of the (e) cecal microbial profile between HF (red) and HF-OFS (blue) rats. Axis indicate the percentage of variation explained by the plotted principal coordinates. Phylum level analysis of the relative abundance in the (f) cecum of HF and HF-OFS rats. Genus, family, and order level analysis of the relative abundance in the (g) cecum of HF and HF-OFS rats. Diverging bar plots show significant Log-Fold Change of bacterial orders, families, and genera in HF-OFS rats compared to HF rats in the (h) cecum. Different taxonomic annotation with the same genus label are appended with numbers in order to separate the values. The feature labels (y-axis) in each plot represent the most specific named taxonomic level describing the feature.

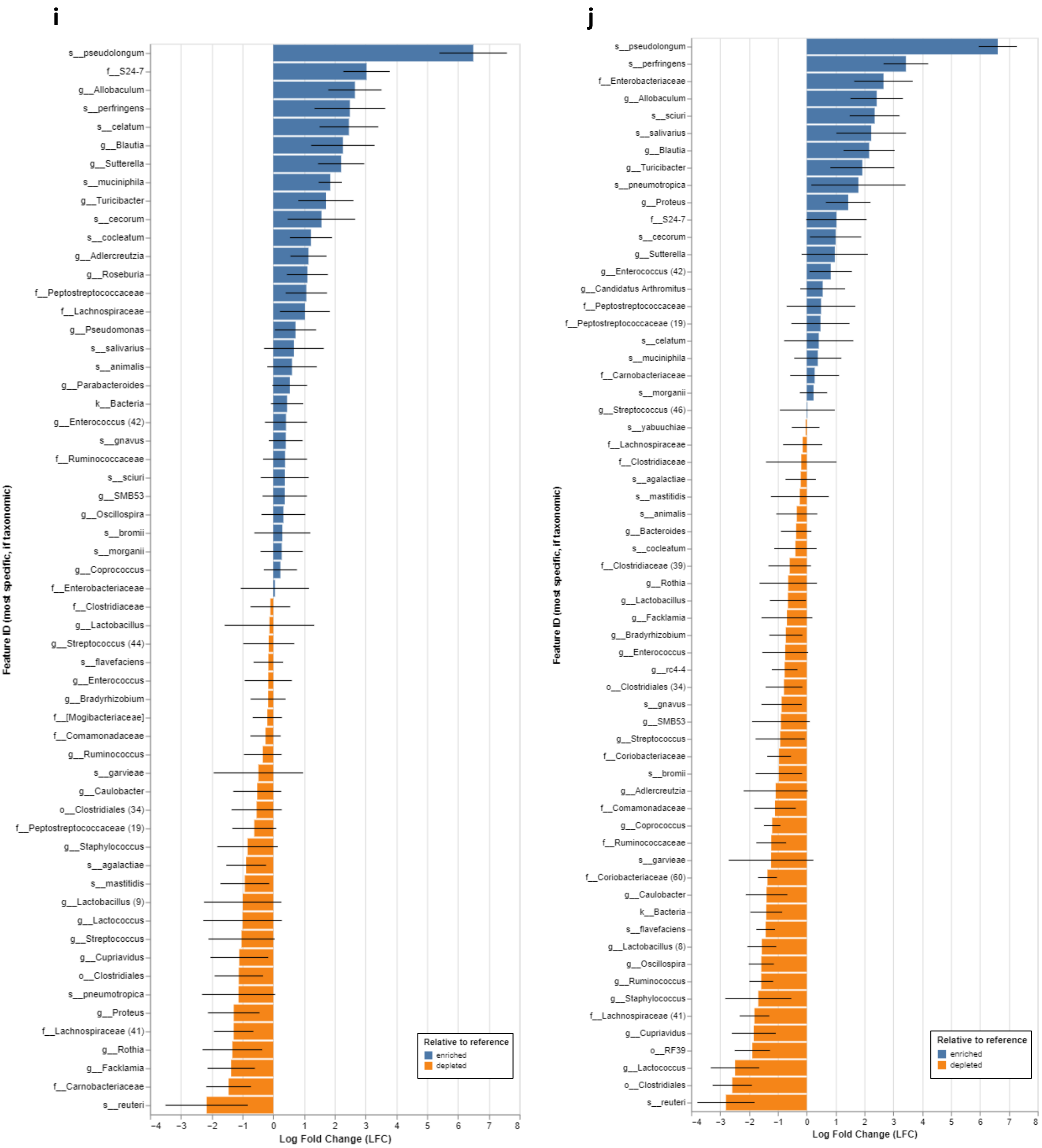

Supplementary Figure 14 (continued). Acute OFS treatment beneficially alters the gut microbiota. Diverging bar plots show significant Log-Fold Change of bacterial orders, families, genera, and species in HF-OFS rats compared to HF rats in the (i) USI and (j) LSI. Different taxonomic annotation with the same genus label are appended with numbers in order to separate the values. The feature labels (y-axis labels) in each plot represent the most specific named taxonomic level describing the feature. Feature identifiers that are duplicated represent instances of a duplicated taxonomic name at the taxonomic level displayed in the feature identifier. The number following the feature identifiers in these cases is used only for unique identification in the current figure. It is not taxonomically meaningful.

k

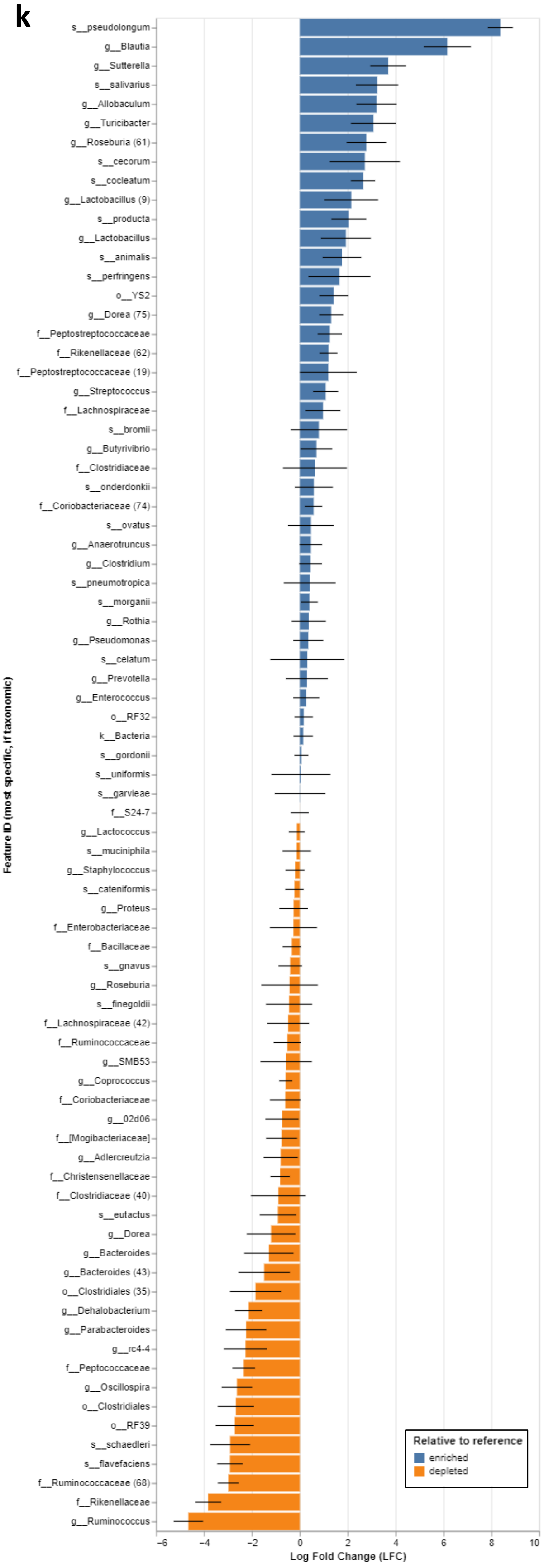

l

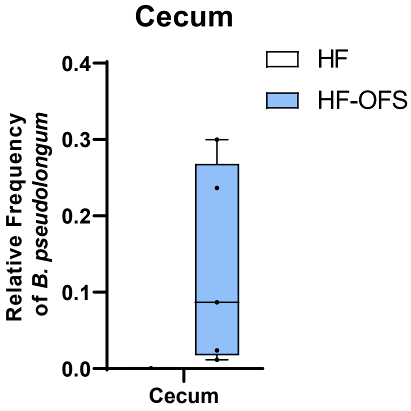

Supplementary Figure 14 (continued). Acute OFS treatment beneficially alters the gut microbiota. Diverging bar plots show significant Log-Fold Change of bacterial orders, families, genera, and species in HF-OFS rats compared to HF rats in the (k) cecum. Different taxonomic annotation with the same genus label are appended with numbers in order to separate the values. The feature labels (y-axis labels) in each plot represent the most specific named taxonomic level describing the feature. Feature identifiers that are duplicated represent instances of a duplicated taxonomic name at the taxonomic level displayed in the feature identifier. The number following the feature identifiers in these cases is used only for unique identification in the current figure. It is not taxonomically meaningful. (l) Box and whisker plots of the relative abundance of *B. pseudolongum* in the cecum of HF and HF-OFS rats.

Supplementary Table 1. Calorie, macronutrient, and micronutrient composition for chow, HF and HF-OFS diets.

	HF Diet (Research Diets D12451)	HF- OFS Diet (Research Diets D19112708)		Chow Diet (Teklad 2018)
**Macronutrients**	***kcal%***	***kcal%***		***kcal%***
Protein	*20.0*	*20.0*		*24.0*
Carbohydrate	*35.0*	*35.0*		*58.0*
Fat	*45.0*	*45.0*		*18.0*
**kcal/gm**	**4.73**	**4.71**		**3.10**
**Ingredients**	***gm%***	***gm%***		
Casein	*23.3%*	*23.2%*		
L-Cystine	*0.3%*	*0.3%*		
Corn Starch	*8.5%*	*4.7%*		
Maltodextrin 10	*11.7%*	*11.6%*		
Sucrose	*20.1%*	*20.0%*		
Cellulose, BW200	*5.8%*	*0.0%*		
Oligofructose	*0.0%*	*10.0%*		
Lard	*20.7%*	*20.6%*		
Soybean Oil	*2.9%*	*2.9%*		
Vitamin Mix V10001	*1.2%*	*1.2%*		
Mineral Mix S10026	*1.2%*	*1.2%*		
**Micronutrients**				
**Vitamin Mix V10001**	***12 g***	***12 g***		***N/A***
Vit A	*4800 IU/kg*	*4800 IU/kg*		*15000 IU/kg*
Vit D3	*1200 IU/kg*	*1200 IU/kg*		*1500 IU/kg*
Vit E	*60 IU/ kg*	*61 IU/ kg*		*110 IU/kg*
Menadione	*0.6 mg/kg*	*0.6 mg/kg*		*50 mg/kg*
Biotin	*0.24 mg/kg*	*0.24 mg/kg*		*0.4 mg/kg*
Vit B12	*12 μg/kg*	*13 μg/kg*		*80 μg/kg*
Folic Acid	*2.4 mg/kg*	*2.4 mg/kg*		*0.4 mg/kg*
Niacin	*36 mg/kg*	*37 mg/kg*		*70 mg/kg*
Pantothenic Acid	*19.2 mg/kg*	*19.2 mg/kg*		*33 mg/kg*
Vit B6	*8.4 mg/kg*	*8.4 mg/kg*		*18 mg/kg*
Vit B2	*7.2 mg/kg*	*7.2 mg/kg*		*15 mg/kg*
Vit B1	*7.2 mg/kg*	*7.2 mg/kg*		*17 mg/kg*
Choline Bitartrate	*2.3 g/kg*	*2.3 g/kg*		*1.2 g/kg*
**Mineral Mix S10026**	***12 g***	***12 g***		***N/A***
Sodium	*1.2 g/ kg*	*1.2 g/ kg*		*0.2%*
Chloride	*1.92 g/ kg*	*1.92 g/ kg*		*0.4%*
Magnesium	*0.6 g/ kg*	*0.6 g/ kg*		*0.2%*
Magnesium Sulfate	*0.396 g/ kg*	*0.396 g/ kg*		
Ammonium Molybdate	*1.92 mg/kg*	*1.92 mg/kg*		
Chromium Potassium Sulfate	*2.4 mg/kg*	*2.4 mg/kg*		
Copper	*7.2 mg/kg*	*7.2 mg/kg*		*15 mg/kg*
Iron	*44.4 mg/kg*	*44.4 mg/kg*		*200 mg/kg*
Manganese	*70.8 mg/kg*	*70.8 mg/kg*		*100 mg/kg*
Iodine	*0.24 mg/kg*	*0.24 mg/kg*		*6 mg/kg*
Flouride	*1.08 mg/kg*	*1.08 mg/kg*		
Selenium	*0.192 mg/kg*	*0.192 mg/kg*		*0.23 mg/kg*
Zinc	*34.8 mg/kg*	*34.8 mg/kg*		*70 mg/kg*
Dicalcium Phosphate	*15.1 g/kg*	*15.1 g/kg*		*1.0%*
Calcium Carbonate	*6.4 g/kg*	*6.4 g/kg*		*0.7%*
Potassium Citrate, 1 H2O	*19.2g/kg*	*19.2g/kg*		*0.6%*
